# Phosphorus-Sulfur Heterocycles Incorporating an O-P(S)-O or O-P(S)-S-S-P(S)-O Scaffold: One-Pot Synthesis and Crystal Structure Study

**DOI:** 10.3390/molecules22101687

**Published:** 2017-10-10

**Authors:** Guoxiong Hua, Kate Davidson, David B. Cordes, Junyi Du, Alexandra M. Z. Slawin, J. Derek Woollins

**Affiliations:** EaStCHEM School of Chemistry, University of St Andrews, Fife KY16 9ST, UK; gh15@st-andrews.ac.uk (G.H.); kd45@st-andrews.ac.uk (K.D.); dbc21@st-andrews.ac.uk (D.B.C.); jd83@st-andrews.ac.uk (J.D.); amzs@st-andrews.ac.uk (A.M.Z.S.)

**Keywords:** 2,4-Diferrocenyl-1,3,2,4-diathiadiphosphetane 2,4-disulfide, diols, one-pot reaction, iodine oxidation, phosphorus-sulfur heterocycles

## Abstract

A new one-pot preparative route was developed to synthesize novel organophosphorus-sulfur heterocycles via the reaction of the four-membered ring thionation reagent [2,4-diferrocenyl-1,3,2,4-diathiadiphosphetane 2,4-disulfide (FcLR, a ferrocene analogue of Lawesson’s reagent)] and alkenyl/aryl-diols and I_2_ (or SOCl_2_) in the presence of triethylamine. Therefore, a series of five- to ten-membered heterocycles bearing an O-P(S)-O or an O-P(S)-S-S-P(S)-O linkage were synthesized. The synthesis features a novel application of the multicomponent reaction, providing an efficient and environmentally benign method for the preparation of the unusual phosphorus-sulfur heterocycles. Seven representative X-ray structures confirm the formation of these heterocycles.

## 1. Introduction

Organic disulfides have attracted considerable interest due to their practical applications in biochemistry, pharmacological chemistry, and industrial chemistry as well as their utility as synthetic reagents [1,2]. For instance, disulfides play an important role in the construction of secondary and tertiary polypeptide and protein structures. Furthermore, the formation of unsymmetrical disulfide bonds is required for the synthesis of many biologically active peptides, peptide mimetics [3], prodrugs, and antibiotics [4,5,6]. Disulfides have also been used for the preparation of self-assembled monolayers (SAMs) [7,8] and monolayer-protected clusters (MPCs) with many versatile properties [9,10]. The synthesis and reactivity of compounds containing multi-sulfur linkage have been studied extensively [11,12,13,14,15,16,17]. Many methods have been developed to prepare cyclic polysulfides by using either elemental sulfur or other sulfur-transfer reagents, however, these methods often gave poor yields of the desired products and require extreme precaution [18,19,20]. There are few samples for the synthesis of the sulfur-phosphorus compounds with O-(P)-S-S-(P)-O scaffold. The synthesis of alkyldithiophosphonate salts with O-(P)-S-S-(P)-O scaffold was first reported in 1970 [21]. Lawesson’s reagent reacted directly with ethylene glycol, 2,2-dimethyl-1,3-propanediol and 2,2′-dihydroxybiphenyl and *tert*-butylamine leading to di-*tert*-butylammonium salts, and the latter were further treated with I_2_ to give the corresponding cyclic disulfides [22]. Recently, three eight-membered phosphorus-sulfur heterocycles have been prepared via a one-pot reaction of alkenyl-diol with 2,4-diferrocenyl-1,3,2,4-diathiadiphosphetane 2,4-disulfide (FcLR, a ferrocene analogue of Lawesson’s reagent) [23]. The development of a highly efficient and systematic method for the preparation of phosphorus-sulfur heterocycles incorporating with an -O-P(S)-S-S-P(S)-O- scaffold is still desirable in organic synthesis. Herein, we report the synthesis and characterization of a series of new five- to ten-membered phosphorus-sulfur heterocycles with S-S linkage by treating 2,4-diferrocenyl-1,3,2,4-diathiadiphosphetane 2,4-disulfide (FcLR) with alkenyl-diols or aryl-diols in the presence of triethylamine followed an intramolecular oxidation by I_2_ or SOCl_2_, and seven related single crystal x-ray structures.

## 2. Results and Discussion

### 2.1. Synthesis and Characterization

**Scheme 1:**
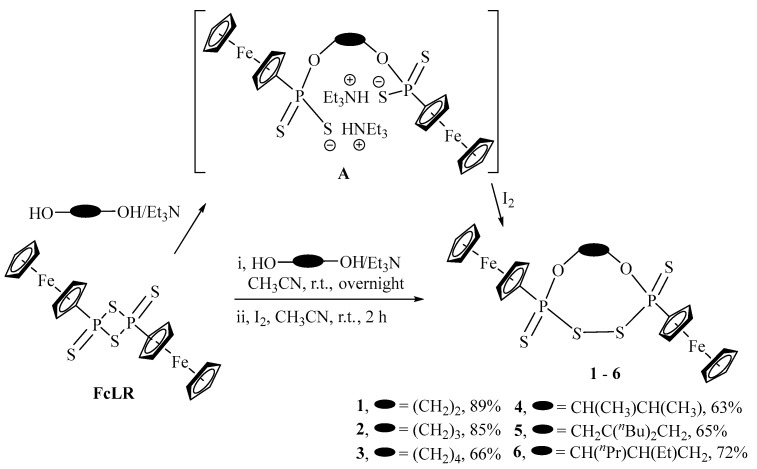
Synthesis of eight- to ten-membered heterocycles **1**–**6** from FcLR, alkenyl-diols and I_2_.

We have reported an efficient route for the synthesis of phosphorus-selenium macrocycles including a series of unusual nine- to fifteen-membered organoselenophosphorus macrocycles by means of Woollins’ reagent reacting with disodium alkenyl-diols, followed by in situ ring-closure reaction with appropriate dihalogenoalkanes [24,25]. A highly efficient route has also been developed successfully by our group for synthesis of a series of nevel twelve- to sixteen-membered organophosphorus-sulfur macrocycles via an one-pot three-component condensation of four-membered ring thionation reagent, 2,4-bis(4-methoxyphenyl)-1,3,2,4-dithiadiphosphetane 2,4-disulfide (LR, Lawesson’s reagent) or 2,4-diferrocenyl-1,3,2,4-diathiadiphosphetane 2,4-disulfide (FcLR), alkenyl-dithiols or aryl-dithiols and dihaloalkanes in the presence of sodium hydride [26]. We adjusted the methods for the preparation of a series of phosphorus-sulfur heterocycles with the O-P(S)-S-S-P(S)-O scaffold. The preparation and spectroscopic characterization of FcLR have been reported by our group [27]. The one-pot reaction of FcLR with an equimolar amount of alkenyl-diol in the presence of two equimolar amounts of triethylamine at room temperature for 24 h, followed by addition of iodine led to the corresponding eight- to ten-membered phosphorus-sulfur heterocycles **1**–**6** in good to excellent yields (63% to 89%), respectively, as depicted in Scheme 1. Insoluble (in water or normal organic solvent) side products which we were not able to identify, but which we assume are linear polymers, were found resulting in the incomplete transformation in all cases. The results show that the reactions tolerate diverse alkenyl-diols with the non-branched or the highly-branched chains, however, the non-branched diols were found to be preferable for the formation of the products in high yields in the current heterocyclization in general, for instance, ethylene glycol gave the product **1** in the highest yield (89%) and the lowest yield (63%) was found in the formation of product **4** in where the butane-2,3-diol was used as starting material. We speculate that **1**–**6** was preferentially formed by intramolecular oxidation/cyclization of intermediate **A** in the presence of two equivalents of triethylamine. 

**Scheme 2:**
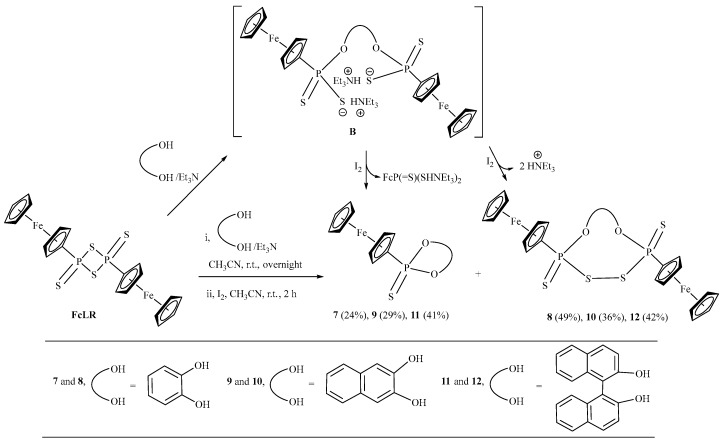
Synthesis of mono-phosphorus species **7**, **9**, **11** and di-phosphorus species **8**, **10**, **12** from FcLR, aryl-diols and I_2_.

Similarly, the reaction of 2,4-diferrocenyl-1,3,2,4-diathiadiphosphetane 2,4-disulfide [FcP(*μ*-S)S]_2_ (FcLR) with an equimolar amount of aryl-diol in the presence of two equimolar amounts of triethylamine at room temperature for 24 h, followed by the oxidation using iodine, gave rise to the additional mono-phosphorus species **7**, **9** and **11** in 24%, 29% and 41% yields respectively together with the expected phosphorus-sulfur heterocycles **8**, **10** and **12** in moderate yields (36 to 49%) as shown in Scheme 2. Once again, in all the cases, insoluble (in water or normal organic solvent) by-products which we were not able to identify were found. The reactions are satisfactory with aryl-diols. The results show that the multiple aromatic ring substitute was preferable to achieve high yields for the formation of both mono-phosphorus species and di-phosphorus heterocycle, for example, when [1,1′-biphenyl]-2,2′-diol is used as diol, the products **11** and **12** were obtained in the highest yields, compared to the other cases. We presume that **7**, **9** and **11** were selectively formed by intramolecular cyclization of intermediate **B** by loss of a molecule of FcP(=S)(SHNEt_3_), and **8**, **10** and **12** were preferentially formed in the same way as compounds **1**–**6**.

**Scheme 3:**
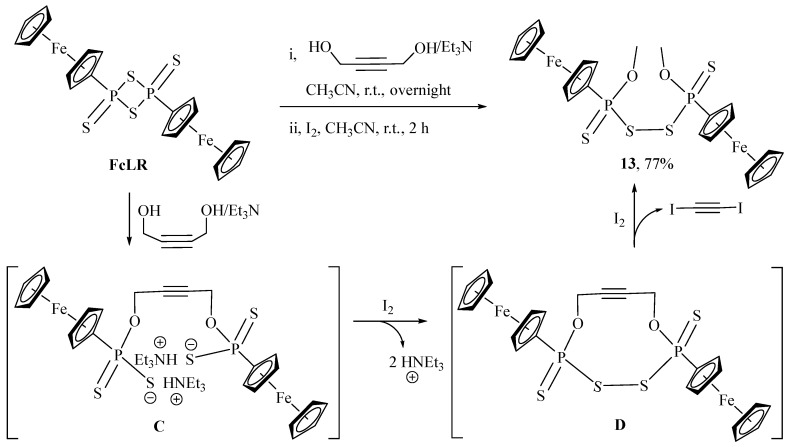
Synthesis of non-heterocycle **13** from FcLR, but-2-yne-1,4-diol and I_2_.

Interestingly, the acyclic product **13** was obtained in 77% yield rather than the expected ten-membered heterocycle **D** when but-2-yne-1,4-diol was used as the starting material under similar reaction conditions (Scheme 3). The preparation and single crystal structure of the disulfide **13** has been reported previously [28]; the compound was synthesized by the iodine oxidation of sodium methoxy(ferrocenyl)dithiophosphonate salt, which was obtained from the reaction of Ferrocenyl Lawesson’s reagent with sodium metal and methanol. However, in that paper, there are no details of characterization and only two diastereomers were found. Here, four diastereomers (ca. 2:1:1:2 intensity ratio) were found in this product due to the two chiral centers present within the molecule. We propose that the heterocyclic compound **D** is formed via an oxidation/cyclisation of the intermediate **C**, however, the heterocycle **D** is not stable and readily decomposed to **13** by the loss of a molecule of IC≡CI in the presence of the oxidant I_2_, further investigation of the mechanism is on the way.

**Scheme 4:**
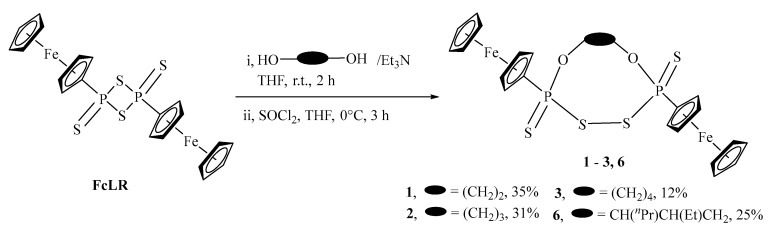
Synthesis of phosphorus-selenium heterocycles **1**–**3**, **6** from FcLR, alkenyl-diols and SOCl_2_.

Surprisingly, attempts to insert a heteroatom into the heterocyclic ring to create a S-S(O)-S linkage failed, and instead the disulfides **1**–**3** and **6** were obtained from the reaction of FcLR with alkenyl-diols, followed by the addition of SOCl_2_ at 0 °C (Scheme 4). Insoluble side products of the reaction were found but we were not able to identify and speculate that they are possibly linear polymers. The average yields of 35%, 31%, 12% and 25% for **1**, **2**, **3** and **6**, respectively, indicate decreasing yield as the size of the ring increases. The results show that the expected insertion of the sulfoxide building block did not happen, instead a disulfide bond was formed in all cases, indicating the thionyl chloride acted as an oxidant to cause the intramolecular cyclisation rather than acting as a ‘building block’ to insert into the heterocyclic ring.

**Figure 1:**
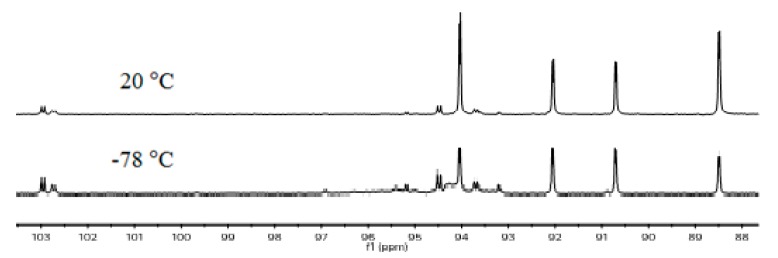
^31^P-NMR spectra of disulfide **6** at the reactions temperatures of 20 °C and −78 °C.

To further investigate the influence of the reaction temperature, the same reaction of FcLR with 2-ethylhexane-1,3-diol and SOCl_2_ was carried out at −78 °C. Heterocycle **6** was obtained as the unique product after work-up and the ^31^P-NMR spectra of two products are shown in Figure 1. Two apparent differences were found with the intensity ratio of four diastereomers changing from ca. 2:1:1:2 to ca. 1:1:1:1 and the yield increasing from 25% to 36%.

The characterization and X-ray structure of compounds **1** [23] and **7** [27] has been reported previously. The characterization of compound **5** has been also reported previously, however, its X-ray structure is not reported yet [22]. The organo-phosphorus-sulfur compounds **2**–**4**, **6**, **8**–**12** are soluble in common polar organic solvents and are air- and moisture-stable at ambient temperature for several months without any apparent decomposition. Standard analytical and spectroscopic techniques were used for the characterization of all the new compounds. There are two potentially stereogenic centers from two phosphorus atoms in compounds **2** and **3**; four potentially stereogenic centers from two phosphorus atoms and two oxygen atoms attached carbon atoms in compounds **4**, **6**, **8**, **10** and **12**. In fact, only one signal is observed in the ^31^P-NMR spectra for the compounds **2**–**4** and **8**, **10** and **12**, however, there are four major diastereomers in ca. 2:1:1:2 intensity ratios in **6** resulting from four chiral centres which lead potentially to 16 diastereomers within the molecule. Compounds **2**–**4**, **6**, **8**–**12** show the anticipated [M]^+^ or [M + H]^+^ peak in their mass spectra and satisfactory accurate mass measurements and appropriate isotopic distributions. The ^1^H-NMR and ^13^C-NMR spectra of compounds **2**–**4**, **6**, **8**–**12** show all the characteristic peaks of the ferrocene backbones. The ^31^P-NMR spectra of di-phosphorus species **2**–**4**, **6**, **8**, **10** and **12** exhibit sharp singlets in the range of δ 88.5 to 95.6 ppm, comparable to the reported values [87.14 to 95.29 ppm] in the similar analogues [22,23]. Meanwhile, compared to di-phosphorus species **2**–**4**, **6**, **8**, **10** and **12**, the ^31^P-NMR spectrum of mono-phosphorus species **9** and **11** show a sharp singlet with the higher chemical shifts of δ 114.1 and 107.8 ppm, respectively, which is similar to the value (113 ppm) in 2-ferrocenylbenzo[*d*][1,3,2]-dioxaphosphole 2-sulfide [27]. The results suggest the influence of the introduction of aryl ring into the rings. Detailed NMR spectroscopic analysis of di-phosphorus species **2**–**4**, **6**, **8**, **10** and **12** reveals the relatively small coupling constant (^3^*J*(P,P) = 3.8 to 4.3 Hz) between two four-coordinate phosphorus centres, supporting the presence of the P-S-S-P group and being consistent with the analogous P-S-S-P system in the literature (cf. ^3^*J*(P,P) = 4 Hz) [22,29]. It should be noted that the chemical shifts of compounds di-phosphorus species **2**–**4**, **6**, **8**, **10** and **12** are clearly different, indicating substantial differences in heterocyclic conformation. 

### 2.2. Single Crystal Structure Analysis

**Figure 2:**
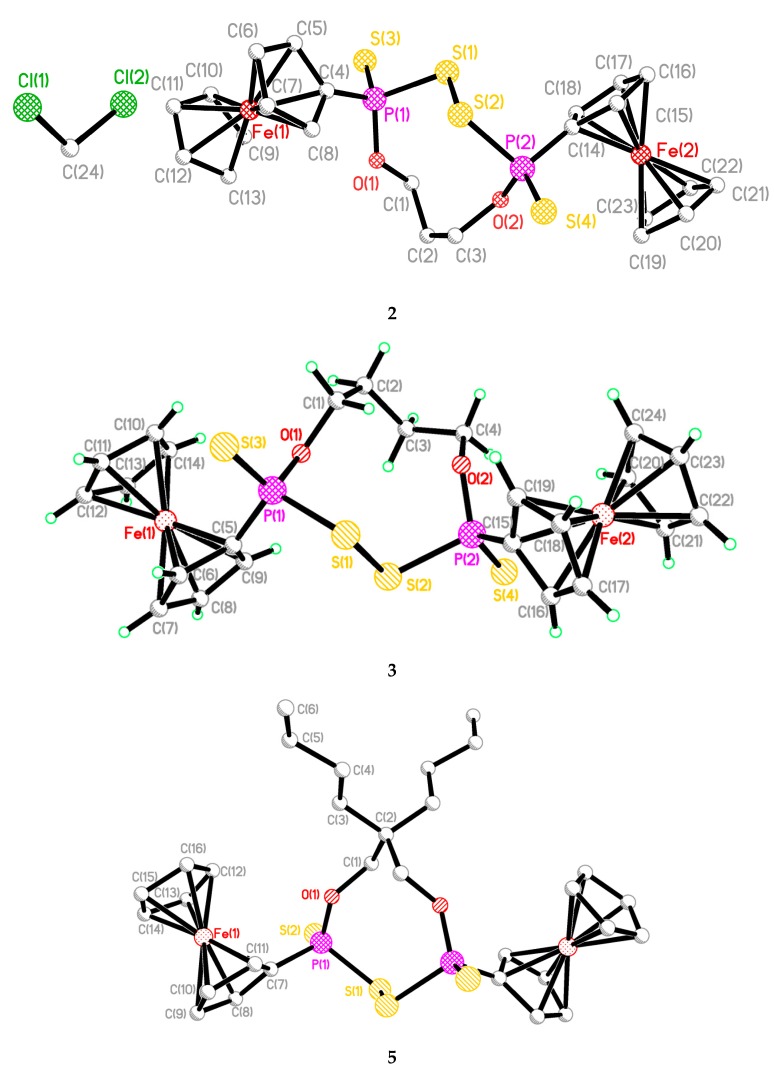

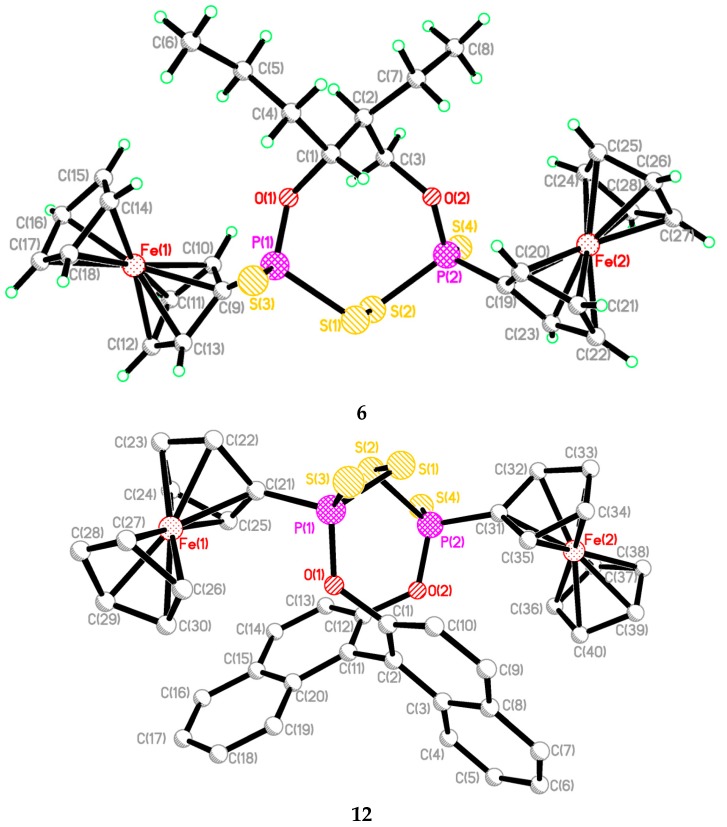
Single crystal X-ray structures of **2**, **3**, **5**, **6** and **12**.

**Figure 3:**
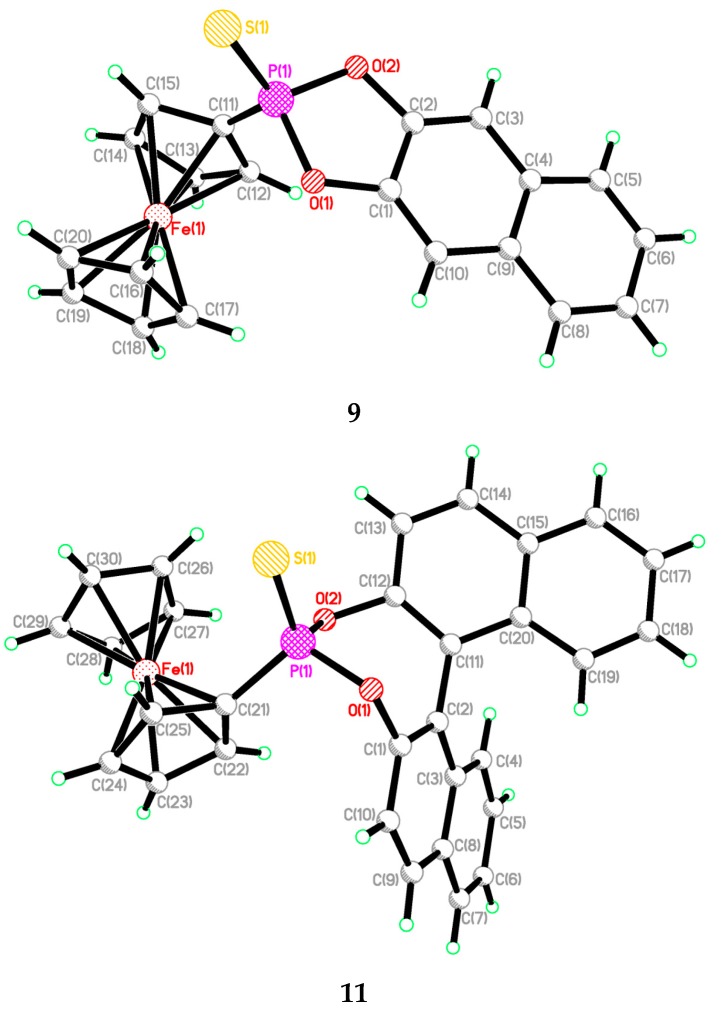
Single crystal X-ray structures of **9** and **11** (hydrogen atoms omitted for clarity).

Crystals of phosphorus-sulfur heterocycles **2**, **3**, **5**, **6**, **9**, **11** and **12** suitable for X-ray crystallographic analysis were grown by diffusion of hexane into a dichloromethane solution of the compound or slow evaporation of a tetrahydrofuran solution of the compound in air at room temperature. The single crystal structures are shown in Figure 2 and Figure 3. The crystallographic data and structure refinement are depicted in Table 1 and Table 2. The selected bond lengths and angles are listed in Table 3 and Table 4. All structures have a single molecule of the compound in the asymmetric unit except **2**, which was found to be co-crystallised with one molecule of chloromethane, and compound **12**, which was co-crystallised with half molecule of sulfur S_8_. The X-ray structures confirm that eight five- to ten-membered rings have formed.

The single crystal structures of **2**, **3**, **5**, **6** and **12** (Figure 2) adopt highly puckered nine- or ten-membered rings with the two ferrocenyl rings pointing towards the top and bottom of the heterocyclic cavity; meanwhile the two exo-cyclic sulfur atoms are *trans* to each other. In addition, all endo-cyclic heteroatoms (O, P, S) are in zigzag position. The angles between the mean plane of two phosphorus atoms and two oxygen atoms (P_2_O_2_), and two adjacent *Cp* rings are 86.93° and 89.95° in **2**, 87.31° and 89.72° in **3**, 88.35° and 88.35° in **5**, 89.02° and 89.08° in **6**, and 79.00° and 89.89° in **12**. The angles between the mean plane of two phosphorus atoms and two oxygen atoms (P_2_O_2_), and two endo-cyclic naphthalene rings are 78.18° and 79.89°, meanwhile the two endo-cyclic naphthalene rings are inclined to almost vertical [85.49°] in **12**. In the solid state, the structure of **5** adopts a more symmetrical conformation than **2**, **3**, **6** and **12**. The transannular P···P distances are 4.521, 4.604, 4.473, 4.510 and 4.673 Å in **2**, **3**, **5**, **6** and **12**, respectively, falling in the ranges that have been reported in the related P-S containing heterocycles [4.97–6.97] [30]. The geometry around P(1) and P(2) is distorted tetrahedral [S(1)-P(1)-S(3) and S(2)-P(2)-S(4): 103.88(8)° and 105.07(7)° in **2**, 104.81(5)° and 103.75(4)]° in **3**, 105.39(3)° and 105.39(3)° in **5**, 102.35(3)° and 104.07(4)° in **6**, and 103.9(2)° and 105.70(19)° in **12**, being smaller than those observed in the P-S containing macrocycles [25] and in related P-Se containing macrocycles [24]. The P-S single bond distances are in the range of 2.093(6) to 2.1087(10) Å and P=S double bond lengths in the range of 1.916(5) to 1.9324(16) Å in the structures of **2**, **3**, **5**, **6** and **12** are marginally shorter than the values [1.934(4) to 1.950(7) Å] for the P=S moiety in phosphorus-sulfur macrocycles [31].

The single crystal structures of **9** and **11** confirm that the five- or seven-membered rings have formed (Figure 3). In the solid state, the structures of **9** and **11** have dihedral angles of 8.54° and 56.13°/60.99° between the mean plane of phosphorus atom and two oxygen atoms (O-P-O) and aryl ring. The angles between the mean plane of phosphorus atom and two oxygen atoms (O-P-O), and the adjacent *Cp* ring are 88.81° and 72.67° in **9** and **11**. The geometry around P is distorted tetrahedral [S(1)-P(1)-O(1) and S(1)-P(1)-O(2): 116.10(7)° and 115.32(7)° in **9**, and 109.46(13)° and 118.65(13)° in **11**], being considerably shorter than that (117.0(2)° in 2-ferrocenylbenzo[*d*][1,3,2]dioxaphosphole 2-sulfide [27], but being much larger than those observed in **2**, **3**, **5**, **6** and **12** and the P-S containing macrocycles [25]. The P=S double bonds in the range of 1.8971(10) Å to 1.9103(17) Å are similar to that (1.893(2) Å) in 2-ferrocenylbenzo[*d*][1,3,2]dioxaphosphole 2-sulfide [27] and marginally shorter than that (1.916(5) to 1,9324(16) Å) in **2**, **3**, **5**, **6** and **12** and significantly shorter than values (1.934(4) to 1.950(7) Å) for the P=S moiety in phosphorus-sulfur macrocycles [31].

## 3. Materials and Methods

### 3.1. General Information

Unless otherwise stated, all reactions were carried out under on oxygen free nitrogen atmosphere using pre-dried solvents and standard Schlenk techniques, subsequent chromatographic and work up procedures were performed in air. ^1^H (400.1 MHz), ^13^C (100.6 MHz), ^31^P-{^1^H} (162.0 MHz) and ^77^Se-{^1^H} (51.4 MHz referenced to external Me_2_Se) NMR spectra were recorded at 25 °C (unless stated otherwise) on Advance II 400s (Bruker, Blue Lion Biotech, Carnation, WA, USA) and GSX 270 (JEOL, Inc., Peabody, MA, USA) spectrometers. IR spectra were recorded as KBr pellets in the range of 4000-250 cm^−1^ on a 2000 FTIR/Raman spectrometer (Perkin-Elmer, Beaconfield, UK). Mass spectrometry was performed by the EPSRC National Mass Spectrometry Service Centre, Swansea. X-ray diffraction data for compound **2** were collected at 125 K using the St Andrews Automated Robotic Diffractometer (STANDARD) [32], consisting of a sealed-tube generator (Rigaku, Houston, USA) equipped with a SHINE monochromator [Mo Kα radiation (λ = 0.71075 Å)], and a Saturn 724 CCD area detector, coupled with a Microglide goniometer head and an ACTOR SM robotic sample changer. Diffraction data for compounds **3**, **5**, **6**, **9**, **11** and **12** were collected at either 93 K (**3**) or 173 K (all other crystals) by using a Rigaku FR-X Ultrahigh Brilliance Microfocus RA generator/confocal optics and Rigaku XtaLAB P200 system, with Mo Kα radiation (λ = 0.71075 Å). Intensity data were collected using ω steps accumulating area detector images spanning at least a hemisphere of reciprocal space. All data were corrected for Lorentz polarization effects. A multi-scan absorption correction was applied by using CrystalClear [33,34]. Structures were solved by either charge-flipping (SUPERFLIP) [35] or direct (SIR2004, SIR2011) [36,37] methods, and refined by full-matrix least-squares against F^2^ (SHELXL-2013) [38]. Non-hydrogen atoms were refined anisotropically, and hydrogen atoms were refined using a riding model. All calculations were performed using the CrystalStructure interface [39]. These data can be obtained free of charge via www.ccdc.cam.ac.uk/conts/retrieving.html or from the Cambridge Crystallographic Data centre, 12 Union Road, Cambridge CB2 1EZ, UK; fax (+44) 1223-336-033; e-mail: deposit@ccdc.cam.ac.uk. CCDC Nos. 1572696–1572702.

### 3.2. Synthesis

#### 3.2.1. General Procedure for the Reaction of FcLR with Alkenyl-diols and I_2_ in the Presence of Triethylamine

A mixture of alkenyl-diol (1.0 mmol) and FcLR (0.56 g, 1.0 mmol) in dry acetonitrile (40 mL) was stirring in the presence of triethylamine (0.202 g, 2.0 mmol) at room temperature overnight. I_2_ solution (0.254 g, 1.0 mmol) in acetonitrile (15 mL) was added dropwise during 2 h and the mixture was continued stirring for another 2 h. Upon removing solvent, the residue was extracted with dichloromethane (20 mL × 3). After removal of the solvent, the crude product was purified by silica column (dichloromethane as eluent) to give the di-phosphorus species **1**–**6**.

*2,5-Diferrocenyl-1,6,3,4,2,5-dioxadithiadiphosphonane 2,5-disulfide* (**2**). Pale yellow solid (0.54 g, 85%). M.p. 189–190 °C. Selected IR (KBr, cm^−1^): 1459(m), 1408(m), 1265(m), 1184(s), 1104(m), 1076(m), 1029(s), 990(s), 972(s), 841(s), 815(s), 751(s), 732(s), 663(s), 543(s), 492(s), 459(m). ^1^H-NMR (400.1 MHz, CDCl_3_, δ), 4.83–4.52 (m, 8H), 4.37 (s, 10H), 4.18 (dt, *J*(P,H) = 10.5 Hz, *J*(H,H) = 5.9 Hz, 4H), 2.26–2.21 (m, 2H) ppm. ^13^C-NMR (100.6 MHz, CDCl_3_, δ), 74.9 (d, *J*(P,C) = 144.5 Hz), 72.6 (d, *J*(P,C) = 11.6 Hz), 71.9 (d, *J*(P,C) = 12.9 Hz), 70.5, 60.5, 28.6 ppm. ^31^P-NMR (162.0 MHz, CDCl_3_, δ), 92.9 ppm. Accurate mass measurement [EI^+^, *m*/*z*]: 633.8833 [M]^+^, calculated mass for C_23_H_24_Fe_2_O_2_P_2_S_4_: 633.8828.

*2,5-Diferrocenyl-1,6,3,4,2,5-dioxadithiadiphosphecane 2,5-disulfide* (**3**). Yellow powder (0.430 g, 66%). M.p. 145–147 °C. Selected IR (KBr, cm^−1^): 1459(m), 1410(m), 1176(s), 1103(m), 1078(m), 1025(m), 993(vs), 922(m), 821(s), 764(s), 664(s), 534(s), 488(vs). ^1^H-NMR (400.1 MHz, CDCl_3_, δ), 4.85–4.48 (m, 8H), 4.34 (s, 10H), 3.89–3.77 (m, 4H), 2.23–2.02 (m, 4H) ppm. ^13^C-NMR (100.6 MHz, CDCl_3_, δ), 76.6 (d, *J*(P,C) = 134.1 Hz), 72.7 (d, *J*(P,C) = 10.2 Hz), 71.6 (d, *J*(P,C) = 22.9 Hz), 70.3, 66.4, 27.4 ppm. ^31^P-NMR (162.0 MHz, CDCl_3_, δ), 92.6 ppm. Accurate mass measurement [EI^+^, *m*/*z*]: 647.8982 [M]^+^, calculated mass for C_24_H_26_Fe_2_O_2_P_2_S_4_: 647.8985.

*7,8-Dimethyl-2,5-diferrocenyl-1,6,3,4,2,5-dioxadithiadiphosphocane 2,5-disulfide* (**4**). Pale yellow solid (0.410 g, 63%). M.p. 63–65 °C. Selected IR (KBr, cm^−1^): 1412(m), 1383(m), 1185(s), 1026(vs), 976(m), 909(s), 823(s), 791(s), 731(s), 669(s), 536(s), 492(s), 474(s). ^1^H-NMR (400.1 MHz, CDCl_3_, δ), 4.81–4.48 (m, 8H), 4.35 (s, 10H), 3.89–3.77 (m, 2H), 1.49 (d, *J*(H,H) = 6.1 Hz, 6H) ppm. ^13^C-NMR (100.6 MHz, CDCl_3_, δ), 77.6 (d, *J*(P,C) = 146.6 Hz), 72.4 (d, *J*(P,C) = 11.9 Hz), 71.6 (d, *J*(P,C) = 12.8 Hz), 70.4, 70.3, 19.5 ppm. ^31^P-NMR (162.0 MHz, CDCl_3_, δ), 90.1 ppm. Accurate mass measurement [CI^+^, *m*/*z*]: 645.9034 [M + H]^+^, calculated mass for C_24_H_25_Fe_2_O_2_P_2_S_4_H: 645.9031.

*8-Ethyl-2,5-diferrocenyl-7-propyl-1,6,3,4,2,5-dioxadithiadiphosphonane 2,5-disulfide* (**6**). Bright yellow solid (0.510 g, 72%). Four diastereoisomers were found in ca. 2:1:1:1:2 intensity ratio in ^31^P-NMR spectra. Selected IR (KBr, cm^−1^): 1459(m), 1411(m), 1386(m), 1365(m), 1184(s), 1107(m), 1024(vs), 972(s), 821(s), 677(vs), 536(s), 487(s). ^1^H-NMR (400.1 MHz, CDCl_3_, δ), 4.83–4.49 (m, 32H , 4.38 (s, 10H), 4.37 (s, 10H), 4.36 (s, 10H), 4.35 (s, 10H), 4.30–4.25 (m, 12H), 2.85–2.76 (m, 1H), 2.73–2.64 (m, 1H), 2.07–1.04 (m, 12H × 4) ppm. ^13^C-NMR (100.6 MHz, CDCl_3_, δ), 77.4 (d, *J*(P,C) = 109 Hz), 77.3 (d, *J*(P,C) = 108 Hz), 75.8 (d, *J*(P,C) = 133 Hz), 74.4 (d, *J*(P,C) = 133 Hz), 72.7 (d, *J*(P,C) = 16.0 Hz), 72.5 (d, *J*(P,C) = 14.4 Hz), 72.4 (d, *J*(P,C) = 10.0 Hz), 72.3 (d, *J*(P,C) = 13.5 Hz), 72.1 (d, *J*(P,C) = 18.6 Hz), 72.0 (d, *J*(P,C) = 19.0 Hz), 71.8 (d, *J*(P,C) = 21.0 Hz), 71.4 (d, *J*(P,C) = 22.9 Hz), 70.6, 70.5, 70.4, 70.4, 64.1 (d, *J*(P,C) = 9.0 Hz), 61.3 (d, *J*(P,C) = 8.6 Hz), 42.1 (t, *J*(P,C) = 14.5 Hz), 41.0 (t, *J*(P,C) = 14.0 Hz), 34.3, 34.1, 19.7, 18.7, 17.4, 15.4, 14.4, 14.0, 12.4, 11.5 ppm. ^31^P-NMR (162.0 MHz, CDCl_3_, δ), 94.0 (d, *J*(P,P) = 4.3 Hz), 92.0 (d, *J*(P,P) = 4.7 Hz), 90.7 (d, *J*(P,P) = 4.7 Hz), 88.5 (d, *J*(P,P) = 4.7 Hz) ppm. Accurate mass measurement [EI^+^, *m*/*z*]: 703.9614 [M]^+^, calculated mass for C_28_H_34_Fe_2_O_2_P_2_S_4_: 703.9610.

#### 3.2.2. General Procedure for the Reaction of FcLR with Aryl-diols (pyrocatechol, naphthalene-2,3-diol, [1,1′-binaphthalene]-2,2′-diol) and I_2_

A mixture of aryl-diol (1.0 mmol) and FcLR (0.56 g, 1.0 mmol) in dry acetonitrile (40 mL) was stirred in the presence of triethylamine (0.202 g, 2.0 mmol) at room temperature overnight. I_2_ solution (0.254 g, 1.0 mmol) in acetonitrile (15 mL) was added dropwise during 2 h and the mixture was continued stirring for another 2 h. Upon removal of the solvent, the residue was extracted with dichloromethane ( 20 mL × 3). After removal of the solvent, the crude product was purified by silica column (dichloromethane as eluent) to give the mono-phosphorus species **7**, **9** and **11**, and di-phosphorus species **8**, **10** and **12**.

*2,5-Diferrocenylbenzo[g][1,6,3,4,2,5]dioxadithiadiphosphocine 2,5-disulfide* (**8**). Yellow paste (0.330 g, 49%). Selected IR (KBr, cm^−1^): 1597(m), 1494(s), 1415(m), 1259(s), 1174(s), 1094(m), 1030(s), 913(s), 819(s), 755(s), 713(m), 664(s), 497(s). ^1^H-NMR (400.1 MHz, CD_2_Cl_2_, δ), 7.15–6.85 (m, 4H), 4.69–4.56 (m, 8H), 4.38 (s, 10H) ppm. ^13^C-NMR (100.6 MHz, CD_2_Cl_2_, δ), 138.5, 126.4, 118.0, 72.2 (d, *J*(P,C) = 177.4 Hz), 72.1 (d, *J*(P,C) = 14.0 Hz), 71.6 (d, *J*(P,C) = 15.5 Hz), 70.5 ppm. ^31^P-NMR (162.0 MHz, CD_2_Cl_2_, δ), 95.5 ppm. Accurate mass measurement [CI^+^, *m*/*z*]: 668.8751 [M + H]^+^, calculated mass for C_26_H_22_Fe_2_O_2_P_2_S_4_H: 668.8754.

*2-Ferrocenylnaphtho[2,3-d][1,3,2]dioxaphosphole 2-sulfide* (**9**). Yellow paste (0.15 g, 29%). Selected IR (KBr, cm^−1^): 1509(m), 1471(s), 1260(s), 1189(s), 1151(m), 1100(s), 1029(s), 927(s), 889(s), 801(s), 744(s), 686(s), 497(s). ^1^H-NMR (400.1 MHz, CD_2_Cl_2_, δ), 7.84 (d, *J*(P,H) = 3.3 Hz, 1H), 7.82 (d, *J*(P,H) = 3.3 Hz, 1H), 7.50–7.47 (m, 4H), 4.60–4.56 (m, 4H), 4.47 (s, 5H) ppm. ^13^C-NMR (100.6 MHz, CD_2_Cl_2_, δ), 144.8, 130.3, 127.4, 126.5, 108.4, 73.2 (d, *J*(P,C) = 14.9 Hz), 72.5 (d, *J*(P,C) = 17.8 Hz), 71.1, 69.8 (d, *J*(P,C) = 160.1 Hz) ppm. ^31^P-NMR (162.0 MHz, CD_2_Cl_2_, δ), 114.1 ppm. Accurate mass measurement [EI^+^, *m*/*z*]: 405.9878 [M]^+^, calculated mass for C_20_H_16_FeO_2_PS: 405.9880.

*2,5-Diferrocenylnaphtho[2,3-g][1,6,3,4,2,5]dioxadithiadiphosphocine 2,5-disulfide* (**10**). Yellow paste (0.259 g, 36%). M.p. 202–204 °C. Selected IR (KBr, cm^−1^): 1513(s), 1471(s), 1410(m), 1366(m), 1265(s), 1189(m), 1115(m), 1103(s), 1031(s), 886(s), 800(s), 743(s), 685(s), 493(s), 455(m). ^1^H-NMR (400.1 MHz, CD_2_Cl_2_, δ), 7.75–7.71 (m, 2H), 7.51–7.36 (m, 4H), 4.73–4.57 (m, 8H), 4.39 (s, 10H) ppm. ^13^C-NMR (100.6 MHz, CD_2_Cl_2_, δ), 146.9, 132.2, 127.1, 126.0, 112.7, 74.2 (d, *J*(P,C) = 104.9 Hz), 72.2 (d, *J*(P,C) = 13.9 Hz), 71.0 (d, *J*(P,C) = 17.4 Hz), 70.6 ppm. ^31^P-NMR (162.0 MHz, CD_2_Cl_2_, δ), 95.6 ppm. Accurate mass measurement [CI^+^, *m*/*z*]: 718.8908 [M + H]^+^, calculated mass for C_30_H_24_Fe_2_O_2_P_2_S_4_H: 718.8911.

*4-Ferrocenyldinaphtho[2,1-d:1′,2′-f][1,3,2]dioxaphosphepine 4-sulfide* (**11**). Pale yellow solid (0.22 g, 41%). M.p. 170–172 °C (Dec.). Selected IR (KBr, cm^–1^): 1586(m), 1502(m), 1467(m), 1409(m), 1538(m), 1209(s), 1184(s), 1071(m), 1026(s), 990(vs), 951(s), 832(s), 814(s), 708(s), 677(m), 650(m), 584(m), 481(s), 437(m). ^1^H-NMR (400.1 MHz, CDCl_3_, δ), 8.86 (d, *J*(H,H) = 9.0 Hz, 2H), 8.27–7.08 (m, 10H), 4.58–4.44 (m, 4H), 4.39 (s, 5H) ppm. ^13^C-NMR (100.6 MHz, CDCl_3_, δ), 134.0, 131.3, 130.4 (d, *J*(P,C) = 21.4 Hz), 129.9, 128.5, 127.7, 127.0 (d, *J*(P,C) = 14.7 Hz), 126.5 (d, *J*(P,C) = 5.8 Hz), 126.0, 119.5, 73.9 (d, *J*(P,C) = 107.9 Hz), 72.5 (d, *J*(P,C) = 15.7 Hz), 71.9 (d, *J*(P,C) = 20.0 Hz), 70.9 ppm.^31^P-NMR (162.0 MHz, CDCl_3_, δ), 107.8 ppm. Accurate mass measurement [EI^+^, *m*/*z*]: 532.0341 [M]^+^, calculated mass for C_30_H_21_FeO_2_PS: 532.0344.

*8,11-Diferrocenyldinaphtho[2,1-g:1′,2′-i][1,6,3,4,2,5]dioxadithiadiphosphecine 8,11-disulfide* (**12**). Pale yellow solid (0.35 g, 42%). M.p. 238–240 °C. Selected IR (KBr, cm^−1^): 1587(m), 1543(m), 1460(m), 1409(m), 1539(m), 1321(m), 1224(s), 1184(s), 1070(s), 1024(s), 988(s), 952(s), 833(s), 753(s), 728(s), 706(m), 680(m), 650(m), 587(m), 484(s). ^1^H-NMR (400.1 MHz, CDCl_3_, δ), 8.89 (d, *J*(H,H) = 9.1 Hz, 2H), 8.22–7.06 (m, 10H), 4.90–4.28 (m, 8H), 4.38 (s, 10H) ppm. ^13^C-NMR (100.6 MHz, CDCl_3_, δ), 134.0, 131.3, 130.4, 129.9, 128.6, 127.7, 127.2 (d, *J*(P,C) = 14.8 Hz), 126.5, 125.6, 119.5, 74.0 (d, *J*(P,C) = 113.1 Hz), 72.3 (d, *J*(P,C) = 15.8 Hz), 71.8 (d, *J*(P,C) = 23.8 Hz), 70.9 ppm.^31^P-NMR (162.0 MHz, CDCl_3_, δ), 92.0 ppm. Accurate mass measurement [EI^+^, *m*/*z*]: 843.9308 [M]^+^, calculated mass for C_40_H_30_Fe_2_O_2_P_2_S_4_: 843.9303.

#### 3.2.3. Synthesis of Compound **13**

A mixture of but-2-yne-1,4-diol (0.086 g, 1.0 mmol) and FcLR (0.560 g, 1.0 mmol) in dry acetonitrile (40 mL) was stirred in the presence of triethylamine (0.404 g, 4.0 mmol) at room temperature overnight. I_2_ solution (0.254 g, 1.0 mmol) in acetonitrile (15 mL) was added dropwise over 2 h and the mixture was continued stirring for another 2 h. After removal of the solvent, the residue was extracted with dichloromethane (20 mL × 3). The crude product was purified by silica column (1:1 hexane/dichloromethane as eluent) to give the title compound **13** as dark yellow solid (0.480 g, 77%).

#### 3.2.4. General Procedure for the Reaction of FcLR with Alkenyl-diols and SOCl_2_

A mixture of alkenyl-diol (1.0 mmol) and FcLR (0.56 g, 1.0 mmol) in dry THF (40 mL) was stirred in the presence of triethylamine (0.202 g, 2.0 mmol) at room temperature for 2 h. Then the mixture was cooled down to 0 °C and SOCl_2_ solution (0.120 g, 1.0 mmol) in THF (15 mL) was added dropwise over 1 h and the mixture was continued stirring for another 2 h. After removal of the solvent, the residue was extracted with dichloromethane (3 × 20 mL). The crude product was purified by silica column (dichloromethane as eluent) to give the di-phosphorus species **1** (35%), **2** (31%), **3** (12%) and **6** (25%).

## 4. Conclusions

In summary, we have successfully developed an efficient approach for the synthesis of a series of novel nine- and ten-membered organophosphorus-sulfur heterocycles with P-S-S-P linkage via a one-pot cyclisation reacton of four-membered ring thionation reagent, 2,4-diferrocenyl-1,3,2,4-diathiadiphosphetane 2,4-disulfide (FcLR), alkenyl-diols or aryl-diols and iodine or SOCl_2_ in the presence of triethylamine. In the case of aryl-diol, an additional five-membered monophosphorus species was obtained apart from the expected thw di-phosphorus species. Seven representative X-ray structures confirm the formation of these organophosphorus-sulfur heterocycles. The reported results enrich the library of organophosphorus-sulfur heterocycles and provide an efficient and envirmentally benign approach to the preparation of the unusual up to ten-membered phosphorus-sulfur heterocycles incorporating an P-S-S-P scaffold.

## Figures and Tables

**Table 1:** Details of the x-ray data collections and refinements for compounds **2**, **3**, **5** and **6**.

Compound	2	3	5	6
Formula	C_24_H_26_Cl_2_Fe_2_O_2_P_2_S_4_	C_24_H_26_Fe_2_O_2_P_2_S_4_	C_31_H_40_Fe_2_O_2_P_2_S_4_	C_28_H_34_Fe_2_O_2_P_2_S_4_
*M*	719.26	648.35	746.45	704.46
Crystal system	triclinic	triclinic	monoclinic	triclinic
Space group	*P-1*	*P-1*	*C2/c*	*P-1*
*a*/Å	7.6366(5)	7.6464(11)	26.246(4)	8.8513(18)
*b*/Å	11.7048(8)	13.1117(18)	9.6717(15)	13.173(3)
*c*/Å	16.7834(11)	14.453(2)	13.720(2)	15.034(3)
*Α*	98.035(5)	67.778(8)	90	106.519(2)
*Β*	100.985(6)	83.624(11)	102.900(4)	105.439(3)
*Γ*	99.739(17)	73.660(10)	90	104.131(3)
*U/A*^3^	1428.35(17)	1287.2(3)	3394.8(9)	1519.8(6)
*Z*	2	2	4	2
*µ*/cm^−1^	16.286	15.968	12.214	13.591
Reflections collected	11190	16874	19978	25008
Independent reflections	4976	4632	3098	5552
*R*_int_	0.0625	0.0405	0.0321	0.0355
*R*1	0.0455	0.0289	0.0267	0.0244
*wR*2 [*I* > 2*σ*(*I*)]	0.1145	0.0713	0.0594	0.0575

**Table 2:** Details of the x-ray data collections and refinements for compounds **9**, **11** and **12**.

Compound	9	11	12
Formula	C_20_H_15_FeO_2_PS	C_30_H_21_FeO_2_PS	C_40_H_30_Fe_2_O_2_P_2_S_8_
*M*	406.22	532.38	972.80
Crystal system	triclinic	monoclinic	Triclinic
Space group	*P-1*	*P2_1_/n*	*P-1*
*a*/Å	7.4940(13)	7.8541(16)	10.874(4)
*b*/Å	10.6802(16)	10.884(2)	14.866(3)
*c*/Å	10.8335(18)	27.176(5)	15.261(4)
*α*	90.128(5)	90	62.01(2)
*β*	94.864(5)	92.738(6)	79.51(3)
*γ*	101.765(5)	90	77.78(3)
*U*/*A*^3^	845.6(2)	2320.5(8)	2119.3(11)
*Z*	2	4	2
*µ*/cm^−1^	11.192	8.362	11.882
Reflections collected	13750	27298	25606
Independent reflections	3066	4241	7692
*R*_int_	0.0319	0.1309	0.1469
*R*1	0.0253	0.0586	0.1261
*wR*2 [*I >* 2*σ*(*I*)]	0.1244	0.1236	0.3345

**Table 3:** Selected bond lengths (Å) and angles (˚) (esds in parentheses) for the structures of **2**, **3**, **5**, **6** and **12**.

	2	3	5	6	12
S(1)-P(1)	2.099(2)	2.1026(9)	2.1034(8)	2.1076(8)	2.093(6)
S(2)-P(2)	2.104(2)	2.1087(10)	2.1034(8)	2.1031(8)	2.107(5)
S(3)-P(1)	1.930(2)	1.9324(16)	1.9210(9)	1.9271(7)	1.916(5)
S(4)-P(2)	1.928(2)	1.9255(12)	1.9210(9)	1.9286(7)	1.921(5)
O(1)-P(1)	1.584(3)	1.587(2)	1.5784(14)	1.5846(14)	1.613(7)
O(2)-P(2)	1.581(3)	1.5830(19)	1.5784(14)	1.5828(16)	1.603(8)
S(1)-S(2)	2.072(2)	2.0789(11)	2.0723(9)	2.0739(7)	2.064(4)
S(1)-P(1)-S(3)	103.88(8)	104.81(5)	105.39(3)	102.35(3)	103.9(2)
S(2)-P(2)-S(4)	105.07(7)	103.75(4)	105.39(3)	104.07(4)	105.70 (19)
S(1)-P(1)-O(1)	107.63(13)	107.44(7)	106.92(6)	108.93(6)	107.3(4)
S(2)-P(2)-O(2)	107.80(13)	108.12(7)	106.92(6)	106.20(6)	107.2(4)
S(3)-P(1)-O(1)	118.95(14)	118.77(6)	119.24(8)	119.55(6)	118.7(3)
S(4)-P(2)-O(2)	117.84(15)	118.77(6)	118.91(10)	119.13(5)	117.7(3)
P(1)-S(1)-S(2)	103.96(8)	102.06(4)	103.72(3)	105.11(3)	106.9(2)
P(2)-S(2)-S(1)	102.63(7)	104.42(4)	103.72(3)	102.76(3)	105.18(17)

**Table 4:** Selected bond lengths (Å) and angles (˚) (esds in parentheses) for the structures of **9** and **11**.

	9	11
S(1)-P(1)	1.9034(8)	1.9103(17)
O(1)-P(1)	1.6303(8)	1.606(3)
O(2)-P(1)	1.6328(17)	1.608(3)
O(1)-C(1)	1.390(3)	1.409(5)
O(2)-C(2)	1.390(3)	1.393(5)
S(1)-P(1)-O(1)	116.10(7)	109.46(13)
S(1)-P(1)-O(2)	115.32(7)	118.65(13)
O(1)-P(1)-O(2)	95.56(9)	102.17(16)
P(1)-O(1)-C(1)	110.60(15)	118.4(3)
P(1)-O(2)-C(2)	110.61(15)	120.3(3)

## Supplementary Materials

Supplementary Materials are available online.
