# Supplementary material for: Phosphorus-Sulfur Heterocycles Incorporating an O-P(S)-O or O-P(S)-S-S-P(S)-O Scaffold: One-Pot Synthesis and Crystal Structure Study
Source: Molecules. 2017 Oct 10;22(10):1687. doi: 10.3390/molecules22101687 (PMC6151443; doi:10.3390/molecules22101687)
Supplement: Supplementary file 1 [file molecules-22-01687-s001.pdf]

Supporting Information

Phosphorus-Sulfur Heterocycles Incorporating with O-P(S)-O or O-P(S)-S-S-P(S)-O Scaffold: One-Pot Synthesis and Crystal Structural Study

*Guoxiong Hua, Kate Davidson, David B. Cordes, Junyi Du, Alexandra M. Z. Slawin and J. Derek Woollins**

School of Chemistry, University of St Andrews, Fife, KY16 9ST, UK
*Corresponding author. Tel.: (+44)-1334-463384; email: jdw3@st-and.ac.uk

1. ^{1}H and ^{13}C NMR spectra of compounds **2-4**, **6**, **8-12**

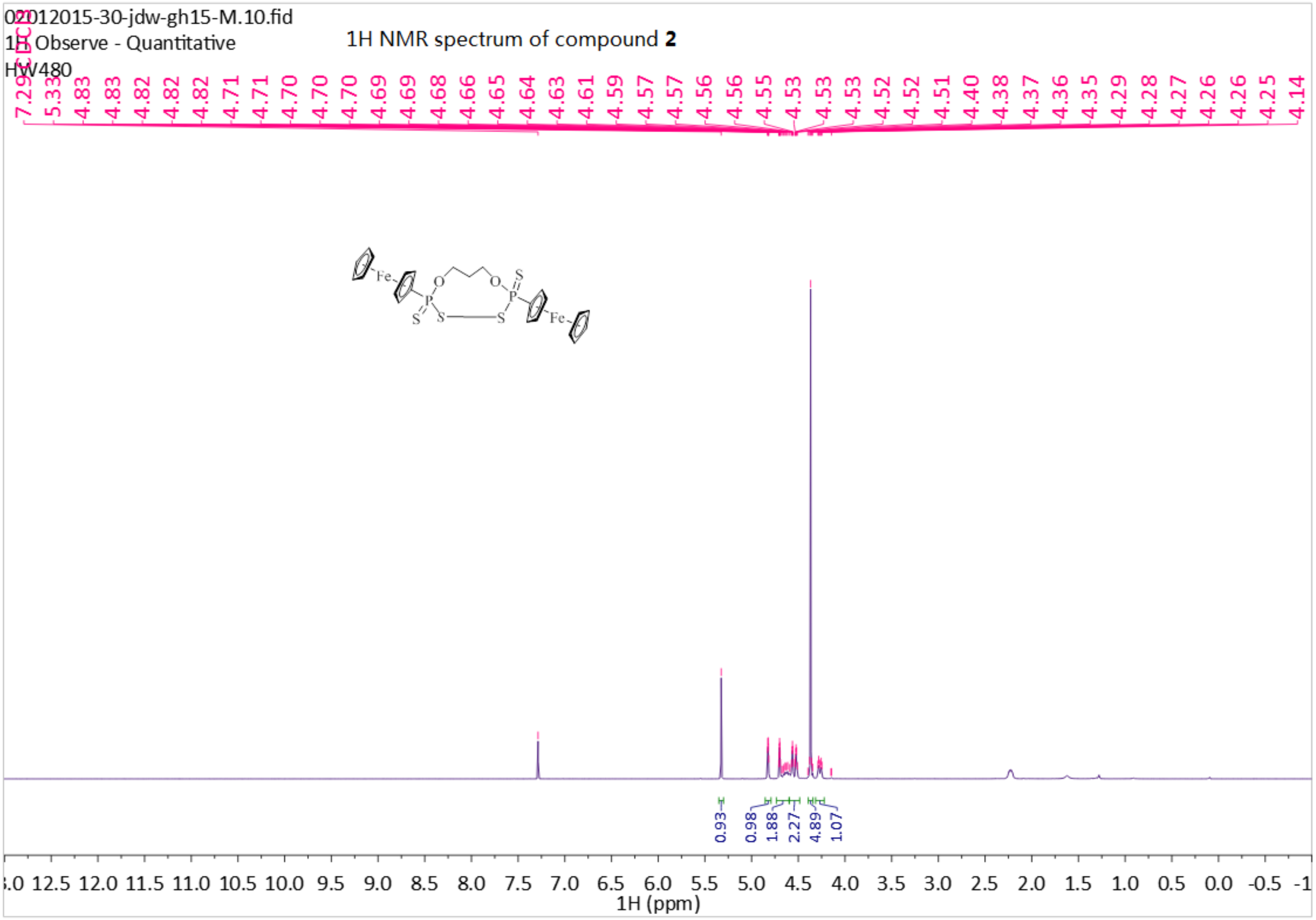

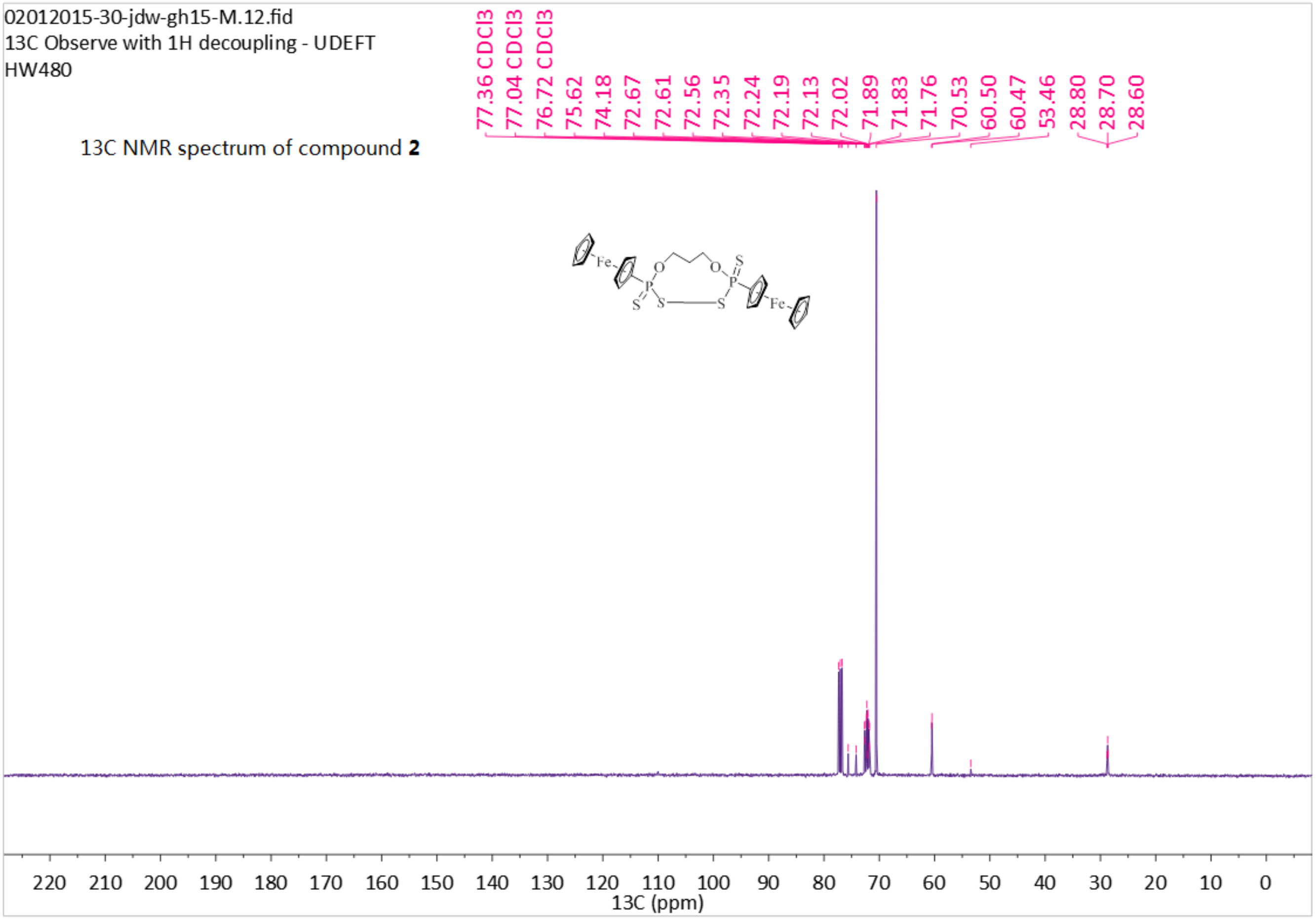

02012015-30-jdw-gh15-M.12.fid
13C Observe with 1H decoupling - UDEFT
HW480
13C NMR spectrum of compound **2**
77.36 CDCl3
77.04 CDCl3
76.72 CDCl3
75.62
74.18
72.67
72.61
72.56
72.35
72.24
72.19
72.13
72.02
71.89
71.83
71.76
70.53
60.50
60.47
53.46
28.80
28.70
28.60
Fe
O
S
P
220 210 200 190 180 170 160 150 140 130 120 110 100 90 80 70 60 50 40 30 20 10 0
13C (ppm)

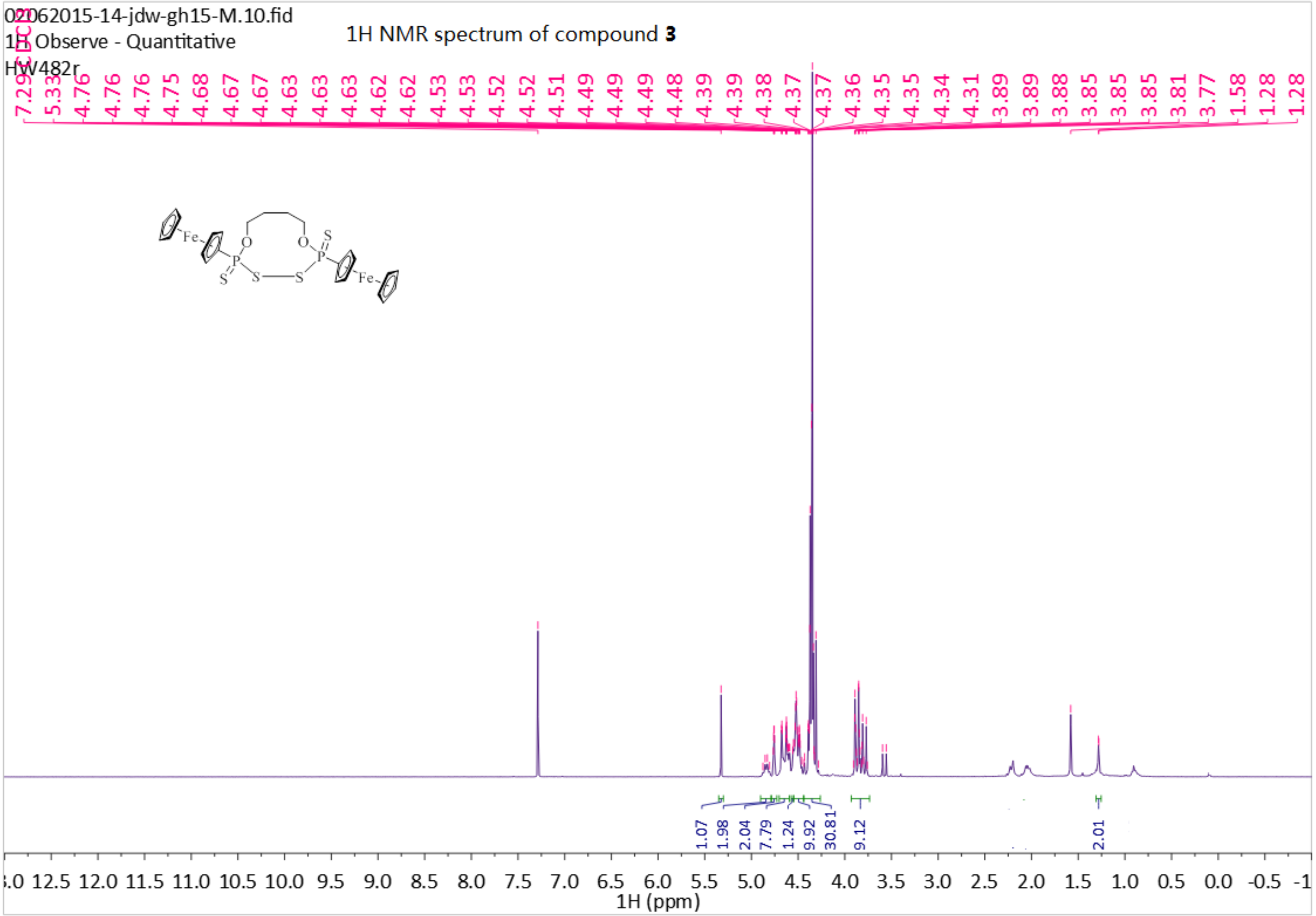

02062015-14-jdw-gh15-M.10.fid
1H Observe - Quantitative
HW482r
1H NMR spectrum of compound **3**
1H (ppm)

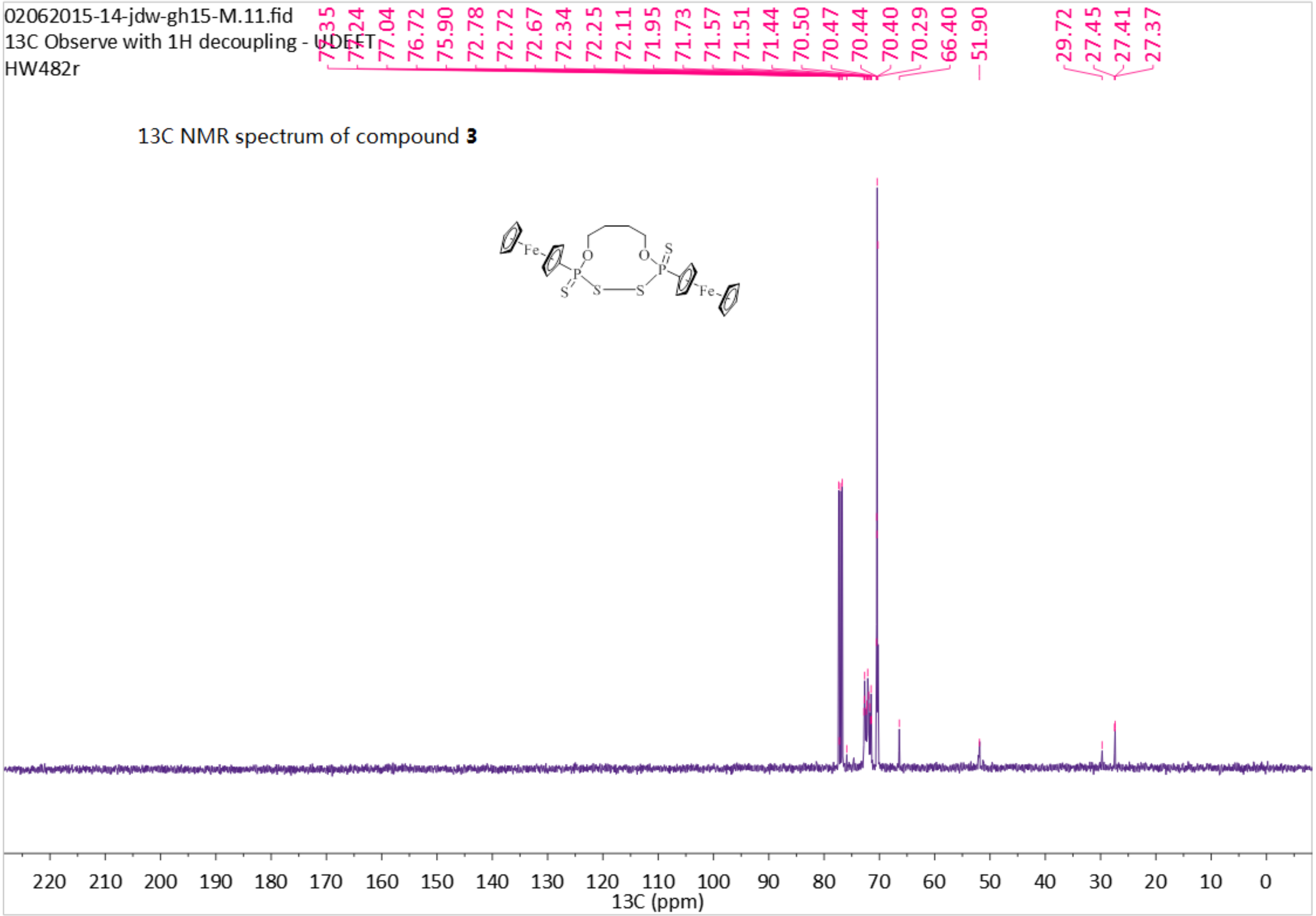
02062015-14-jdw-gh15-M.11.fid
13C Observe with 1H decoupling - UDEFT
HW482r
13C NMR spectrum of compound **3**
77.04
76.72
75.90
72.78
72.72
72.67
72.34
72.25
72.11
71.95
71.73
71.57
71.51
71.44
70.50
70.47
70.44
70.40
70.29
66.40
51.90
29.72
27.45
27.41
27.37
Fe
O
S
P
220 210 200 190 180 170 160 150 140 130 120 110 100 90 80 70 60 50 40 30 20 10 0
13C (ppm)

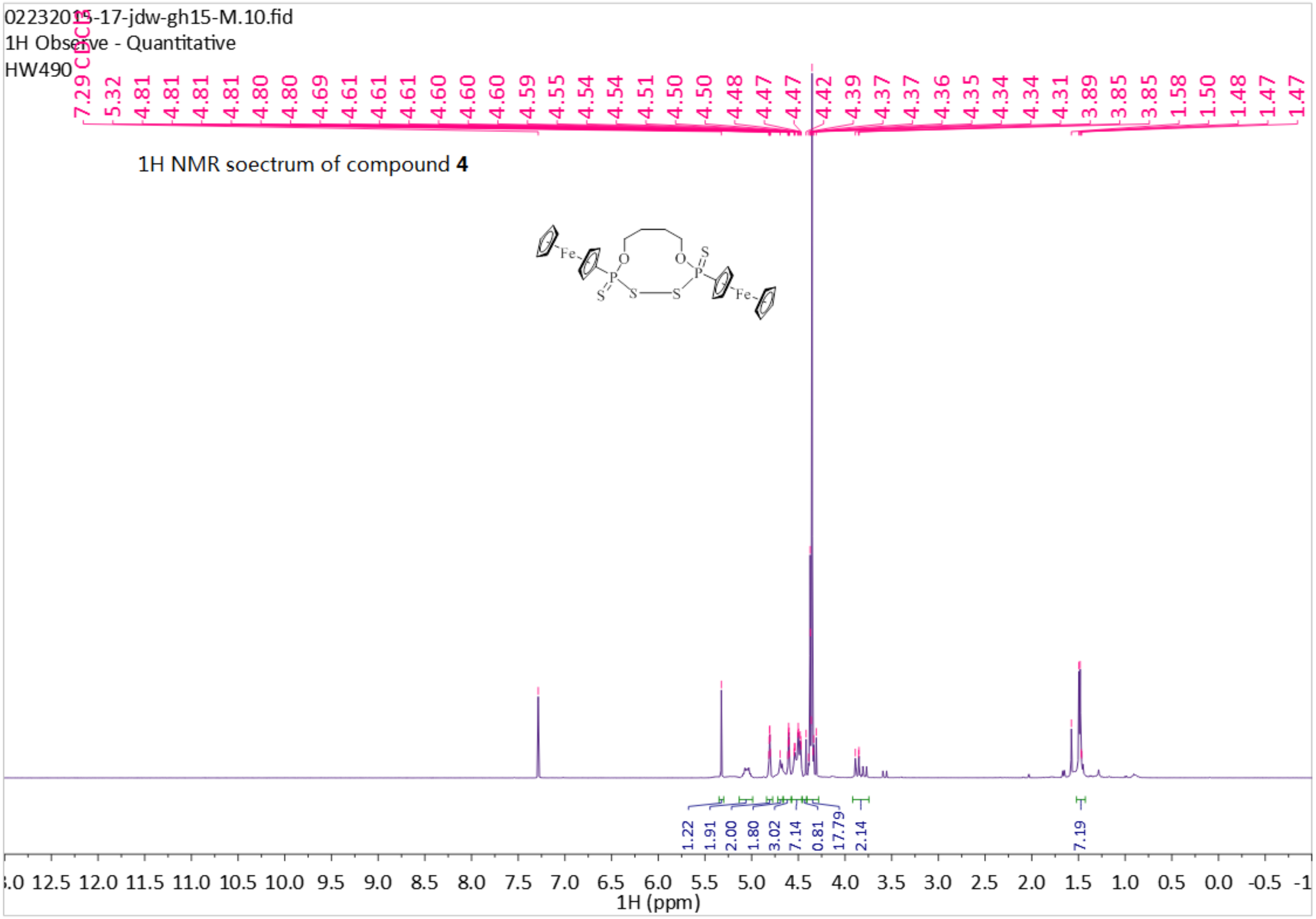
02232015-17-jdw-gh15-M.10.fid
1H Observe - Quantitative
HW490
CDCl3
7.29
5.32
4.81
4.81
4.81
4.81
4.80
4.80
4.69
4.61
4.61
4.61
4.60
4.60
4.60
4.59
4.55
4.54
4.54
4.51
4.50
4.50
4.48
4.47
4.47
4.42
4.39
4.37
4.37
4.36
4.35
4.34
4.34
4.31
3.89
3.85
3.85
1.58
1.50
1.48
1.47
1.47
1H NMR soectrum of compound **4**
1.22
1.91
2.00
1.80
3.02
7.14
0.81
17.79
2.14
7.19
1H (ppm)

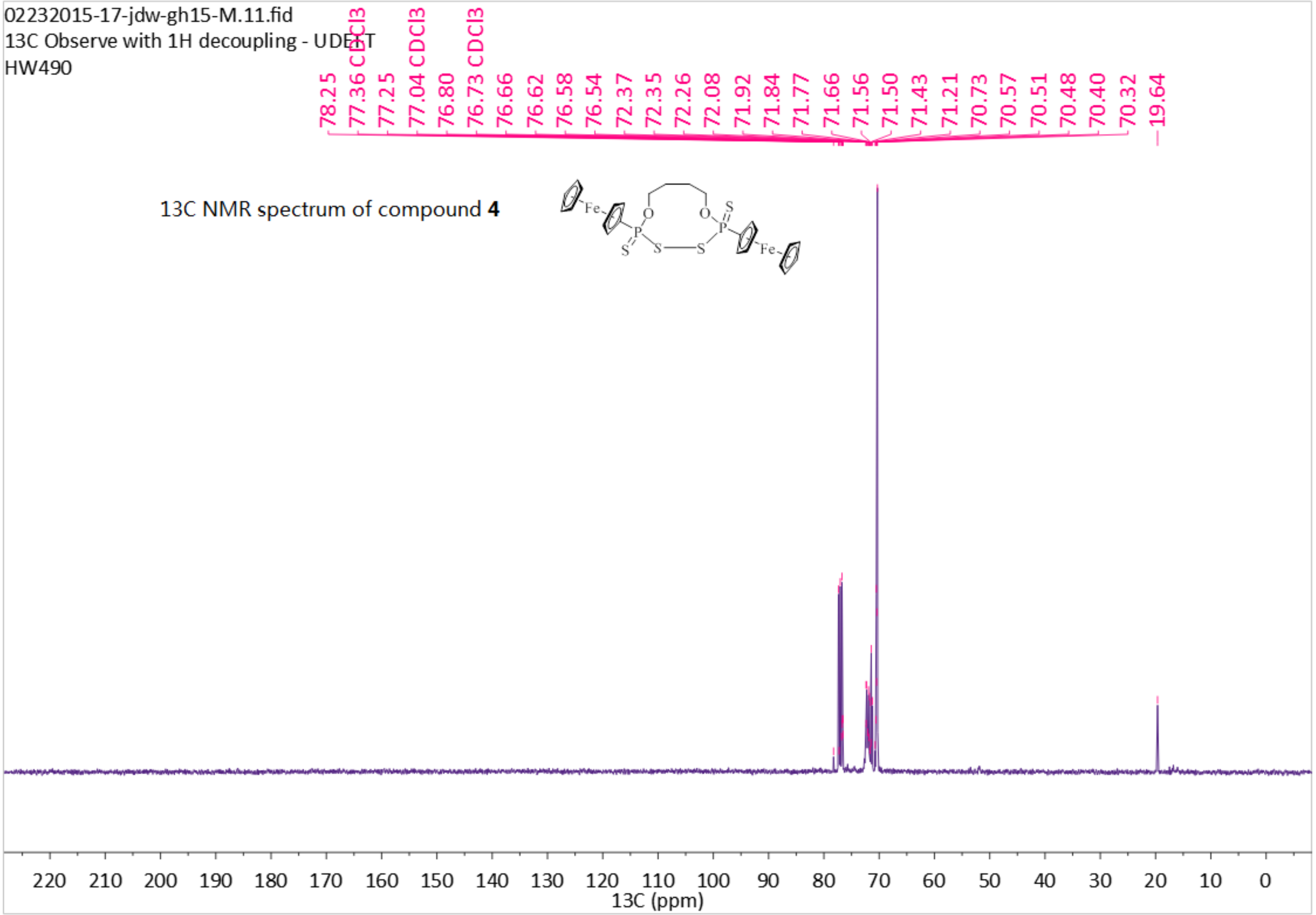
02232015-17-jdw-gh15-M.11.fid
13C Observe with 1H decoupling - UDEFT
HW490
78.25
77.36 CDCl3
77.25
77.04 CDCl3
76.80
76.73 CDCl3
76.66
76.62
76.58
76.54
72.37
72.35
72.26
72.08
71.92
71.84
71.77
71.66
71.56
71.50
71.43
71.21
70.73
70.57
70.51
70.48
70.40
70.32
19.64
13C NMR spectrum of compound 4
Fe
O
O
P
P
S
S
S
S
Fe
220 210 200 190 180 170 160 150 140 130 120 110 100 90 80 70 60 50 40 30 20 10 0
13C (ppm)

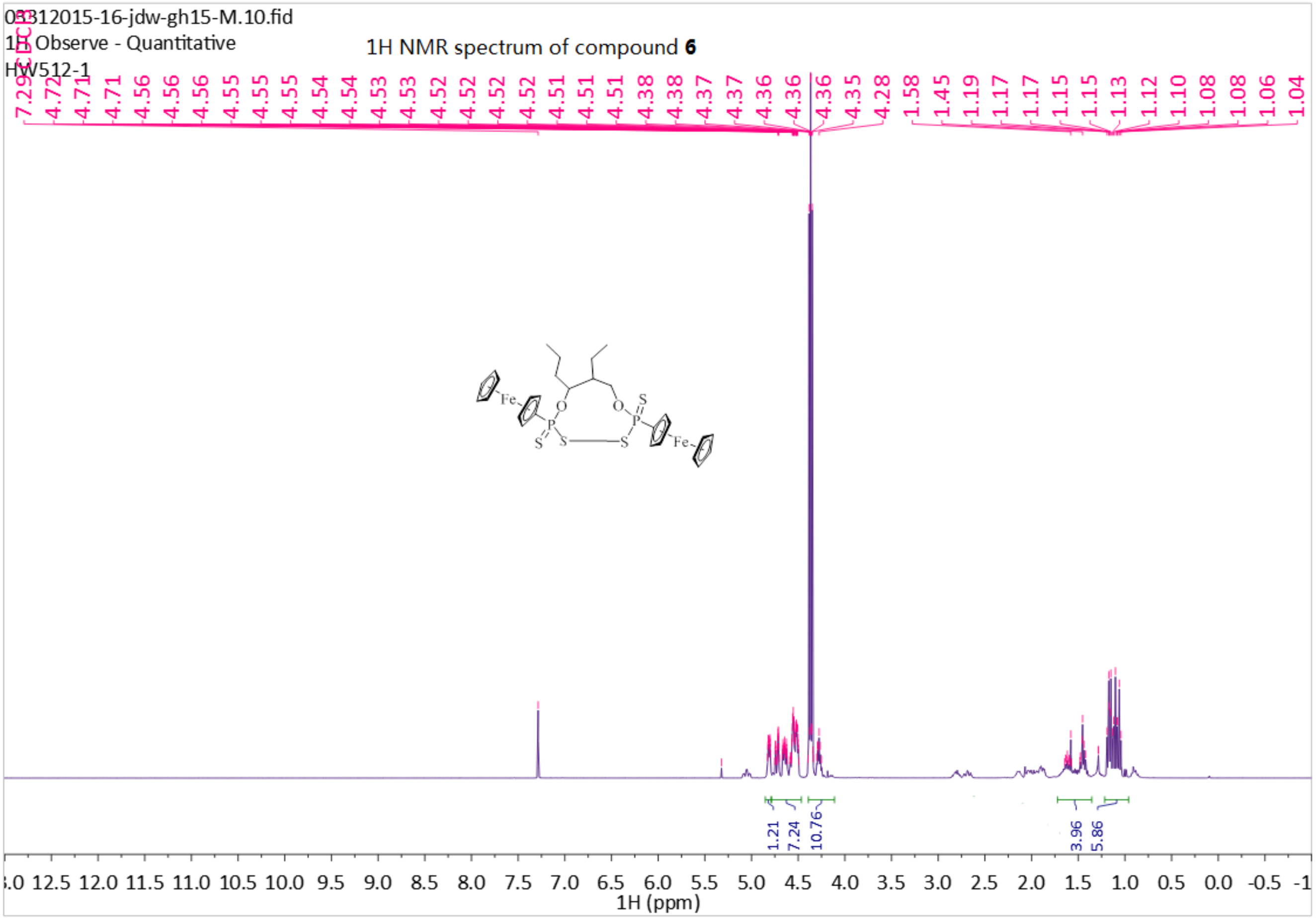

03312015-16-jdw-gh15-M.10.fid
1H Observe - Quantitative
HW512-1
1H NMR spectrum of compound 6
CDCl3
7.29
4.72
4.71
4.71
4.56
4.56
4.56
4.55
4.55
4.55
4.54
4.54
4.53
4.53
4.52
4.52
4.52
4.52
4.51
4.51
4.51
4.38
4.38
4.37
4.37
4.36
4.36
4.36
4.35
4.28
1.58
1.45
1.19
1.17
1.17
1.15
1.15
1.13
1.12
1.10
1.08
1.08
1.06
1.04
Fe
O
P
S
1.21
7.24
10.76
3.96
5.86
1H (ppm)

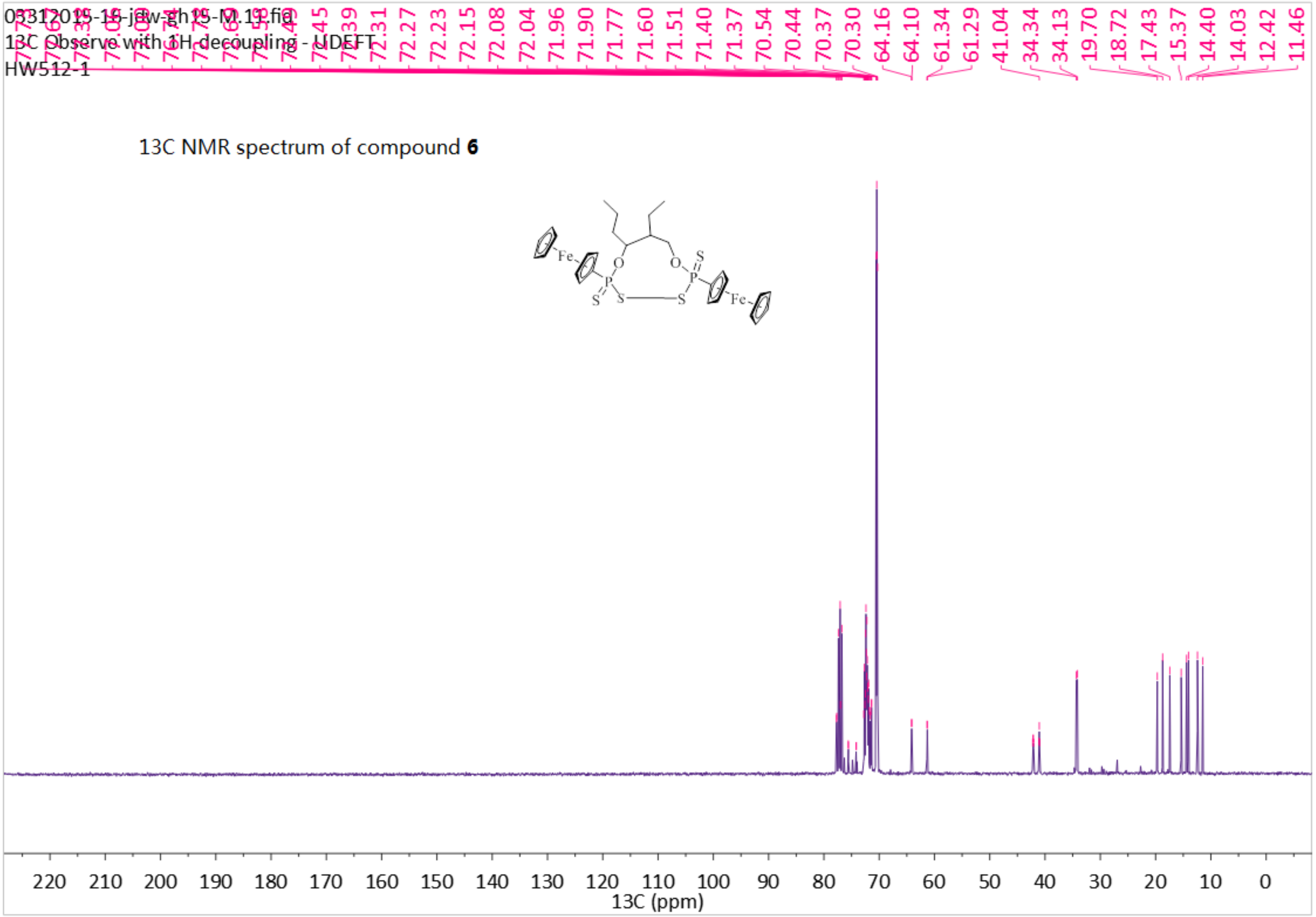
HW512-1
13C NMR spectrum of compound 6
72.45
72.39
72.31
72.27
72.23
72.15
72.08
72.04
71.96
71.90
71.77
71.60
71.51
71.40
71.37
70.54
70.44
70.37
70.30
64.16
64.10
61.34
61.29
41.04
34.34
34.13
19.70
18.72
17.43
15.37
14.40
14.03
12.42
11.46
220 210 200 190 180 170 160 150 140 130 120 110 100 90 80 70 60 50 40 30 20 10 0
13C (ppm)

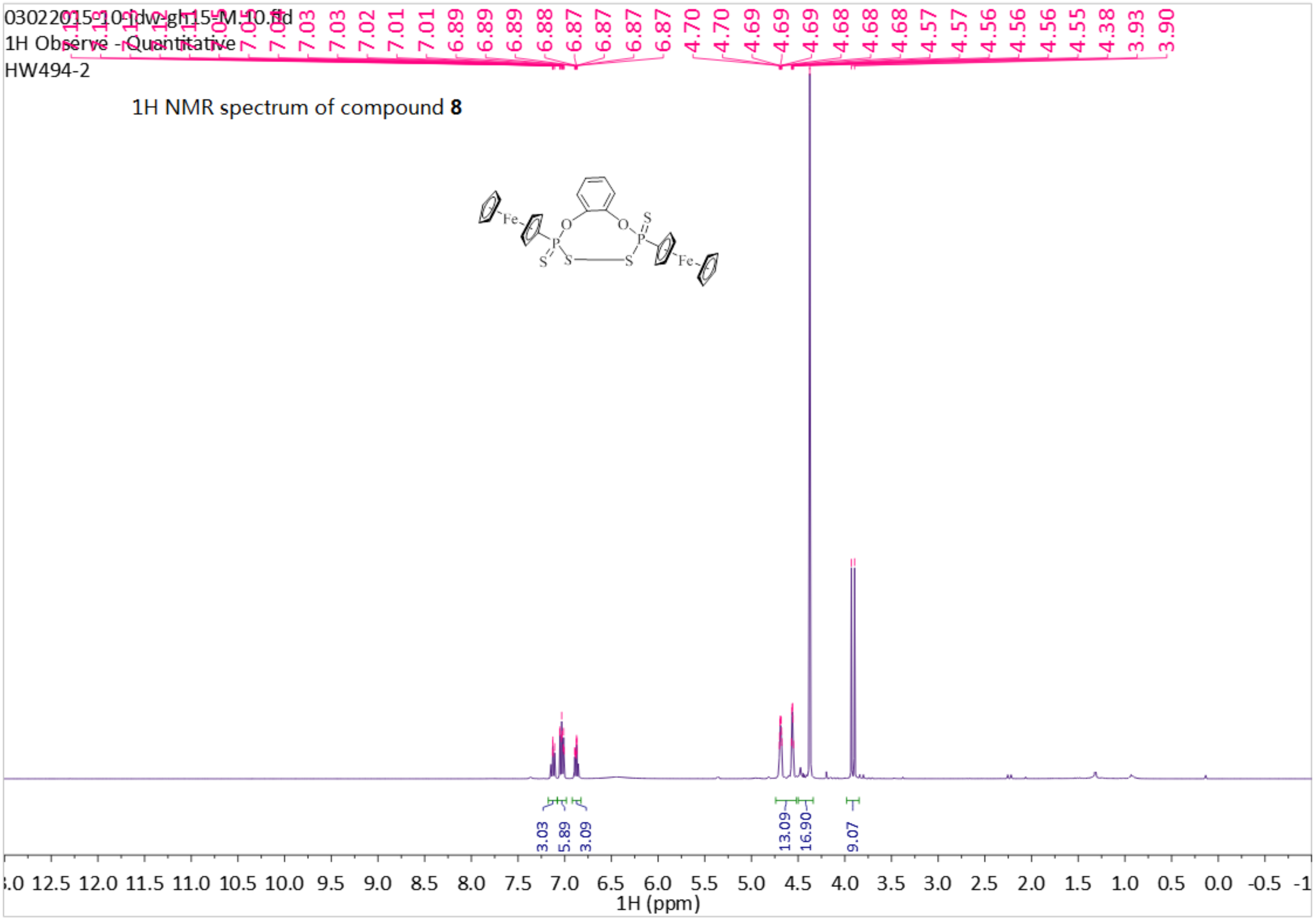
1H Observe - Quantitative
HW494-2
1H NMR spectrum of compound **8**
7.03 7.03 7.02 7.01 7.01 6.89 6.89 6.89 6.88 6.87 6.87 6.87 6.87 4.70 4.70 4.69 4.69 4.69 4.68 4.68 4.68 4.57 4.57 4.56 4.56 4.56 4.55 4.38 3.93 3.90
3.03 5.89 3.09 13.09 16.90 9.07
12.5 12.0 11.5 11.0 10.5 10.0 9.5 9.0 8.5 8.0 7.5 7.0 6.5 6.0 5.5 5.0 4.5 4.0 3.5 3.0 2.5 2.0 1.5 1.0 0.5 0.0 -0.5
1H (ppm)

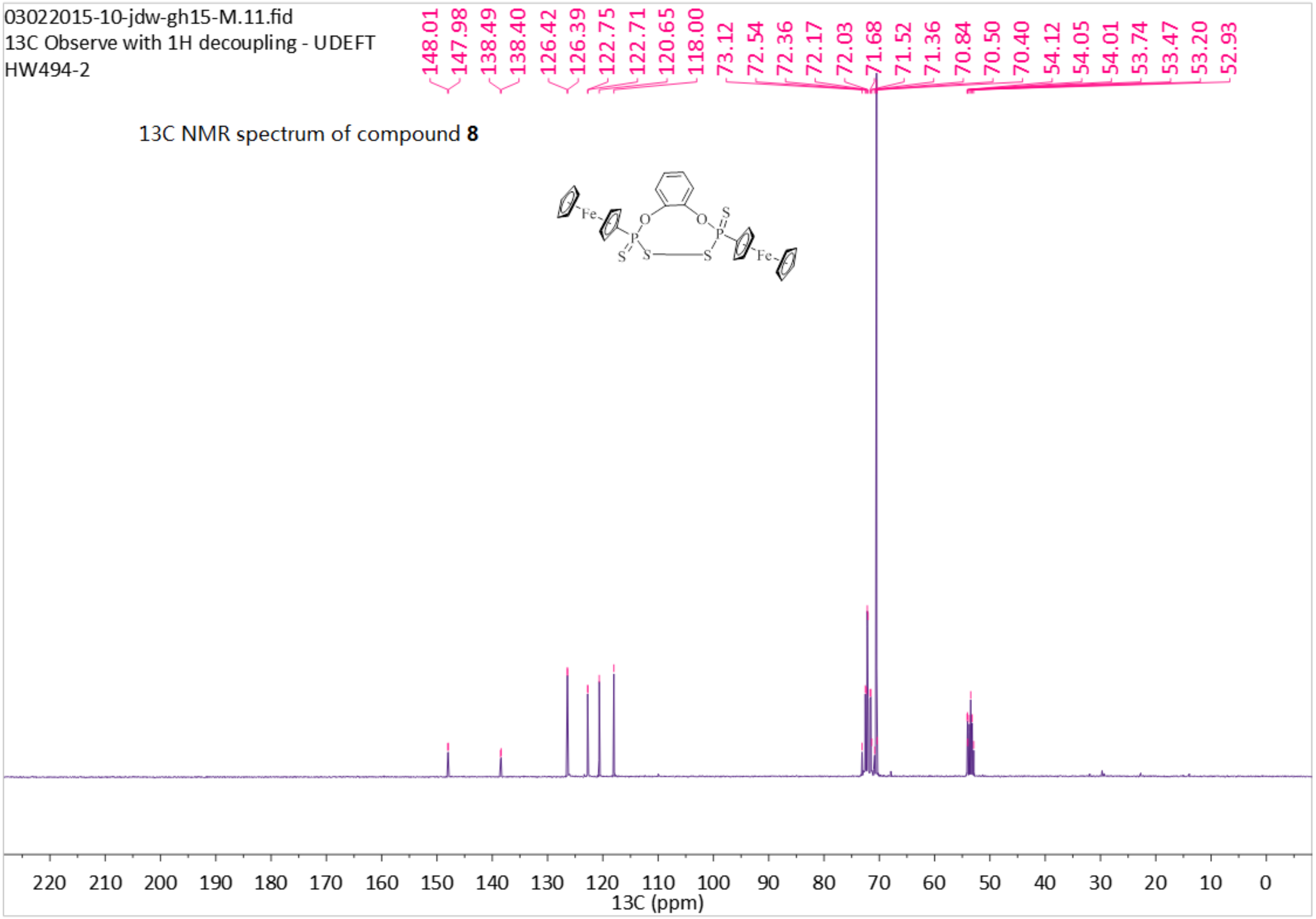
03022015-10-jdw-gh15-M.11.fid
13C Observe with 1H decoupling - UDEFT
HW494-2
148.01
147.98
138.49
138.40
126.42
126.39
122.75
122.71
120.65
118.00
73.12
72.54
72.36
72.17
72.03
71.68
71.52
71.36
70.84
70.50
70.40
54.12
54.05
54.01
53.74
53.47
53.20
52.93
13C NMR spectrum of compound **8**
Fe
O
O
P
P
S
S
S
S
Fe
220 210 200 190 180 170 160 150 140 130 120 110 100 90 80 70 60 50 40 30 20 10 0
13C (ppm)

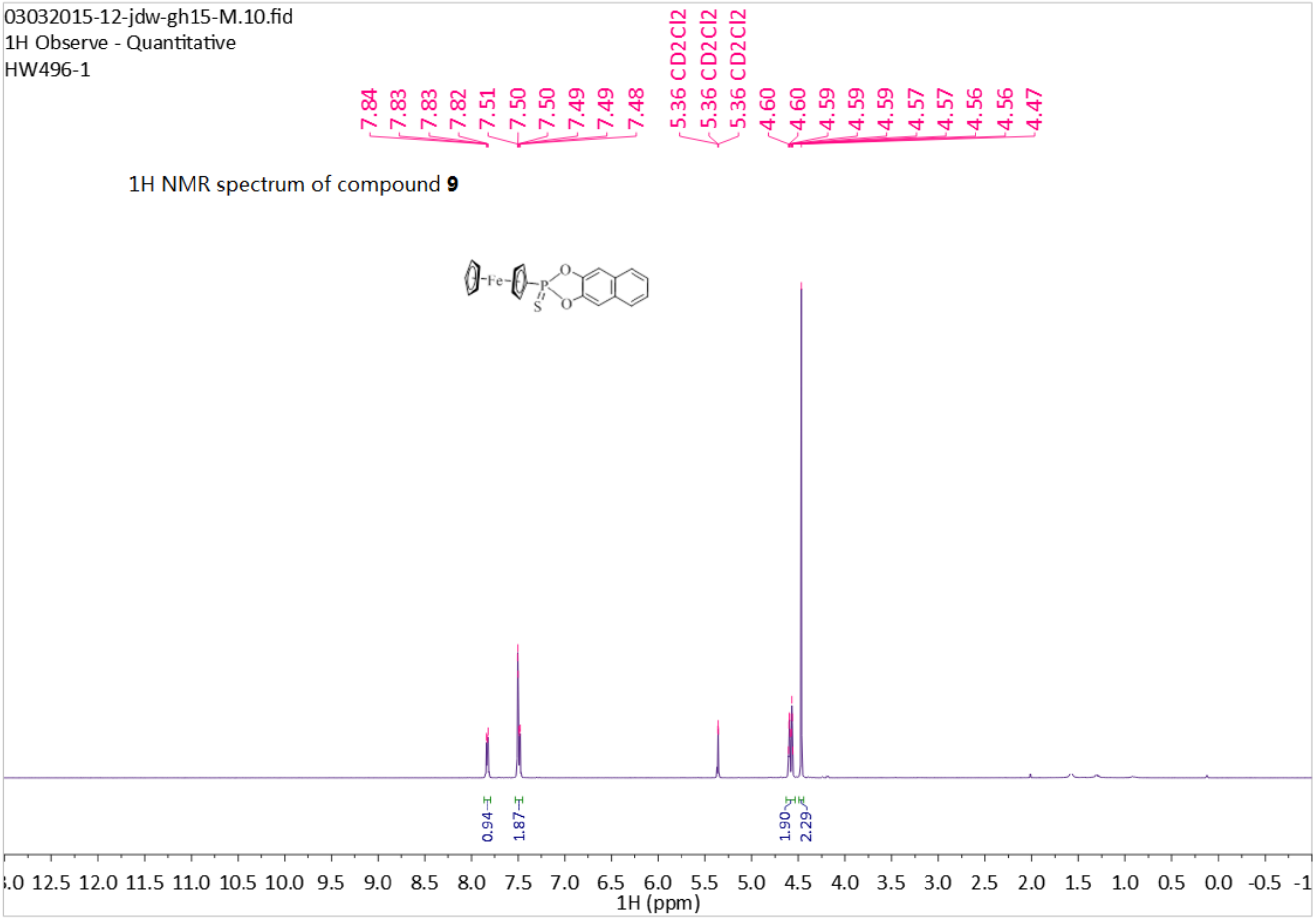
03032015-12-jdw-gh15-M.10.fid
1H Observe - Quantitative
HW496-1
1H NMR spectrum of compound 9
7.84 7.83 7.83 7.82 7.51 7.50 7.50 7.49 7.49 7.48
5.36 CD2Cl2 5.36 CD2Cl2 5.36 CD2Cl2
4.60 4.60 4.59 4.59 4.59 4.57 4.57 4.56 4.56 4.47
0.94
1.87
1.90
2.29
1H (ppm)

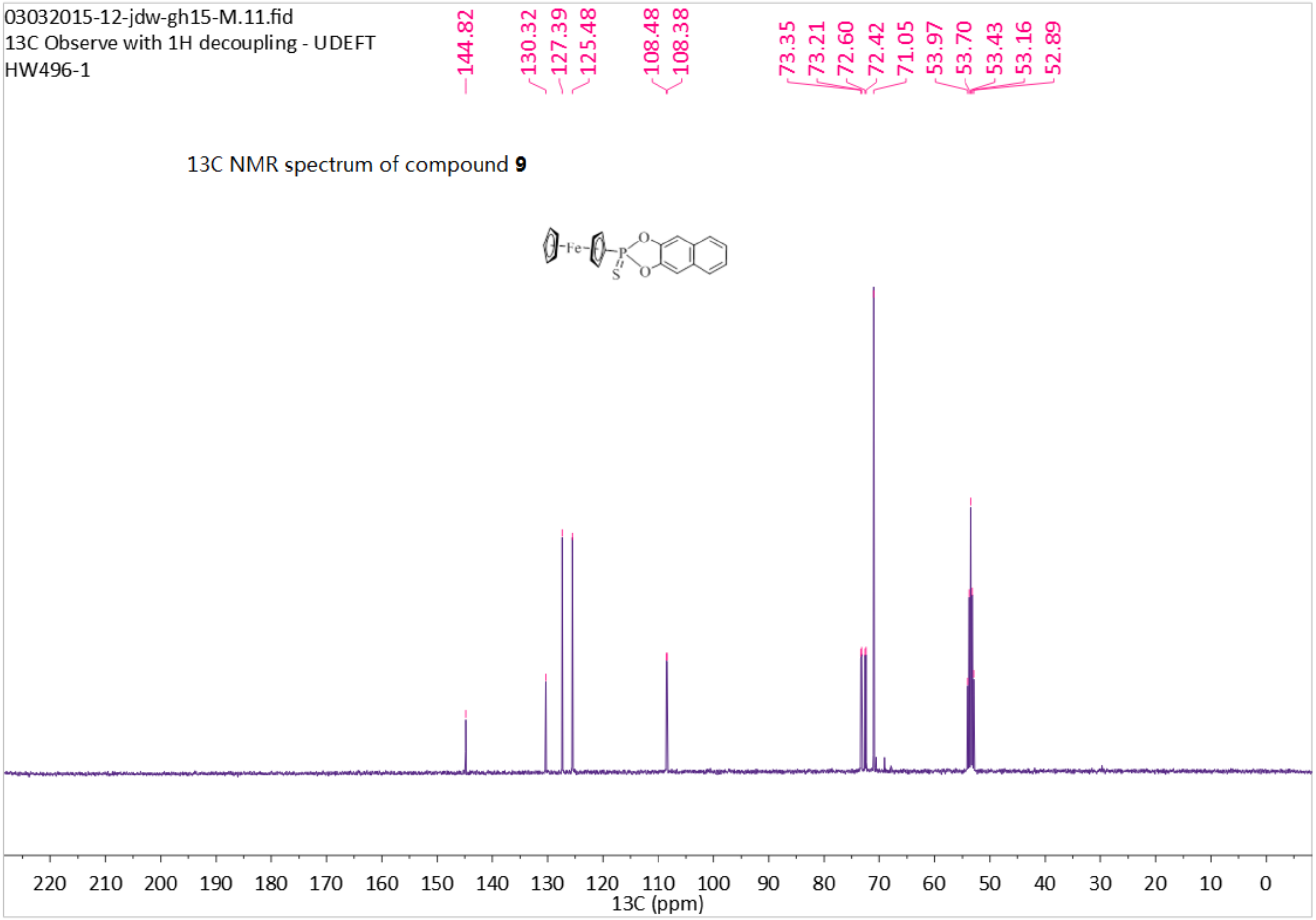
03032015-12-jdw-gh15-M.11.fid
13C Observe with 1H decoupling - UDEFT
HW496-1
144.82
130.32
127.39
125.48
108.48
108.38
73.35
73.21
72.60
72.42
71.05
53.97
53.70
53.43
53.16
52.89
13C NMR spectrum of compound 9
Fe
P
O
O
S
220 210 200 190 180 170 160 150 140 130 120 110 100 90 80 70 60 50 40 30 20 10 0
13C (ppm)

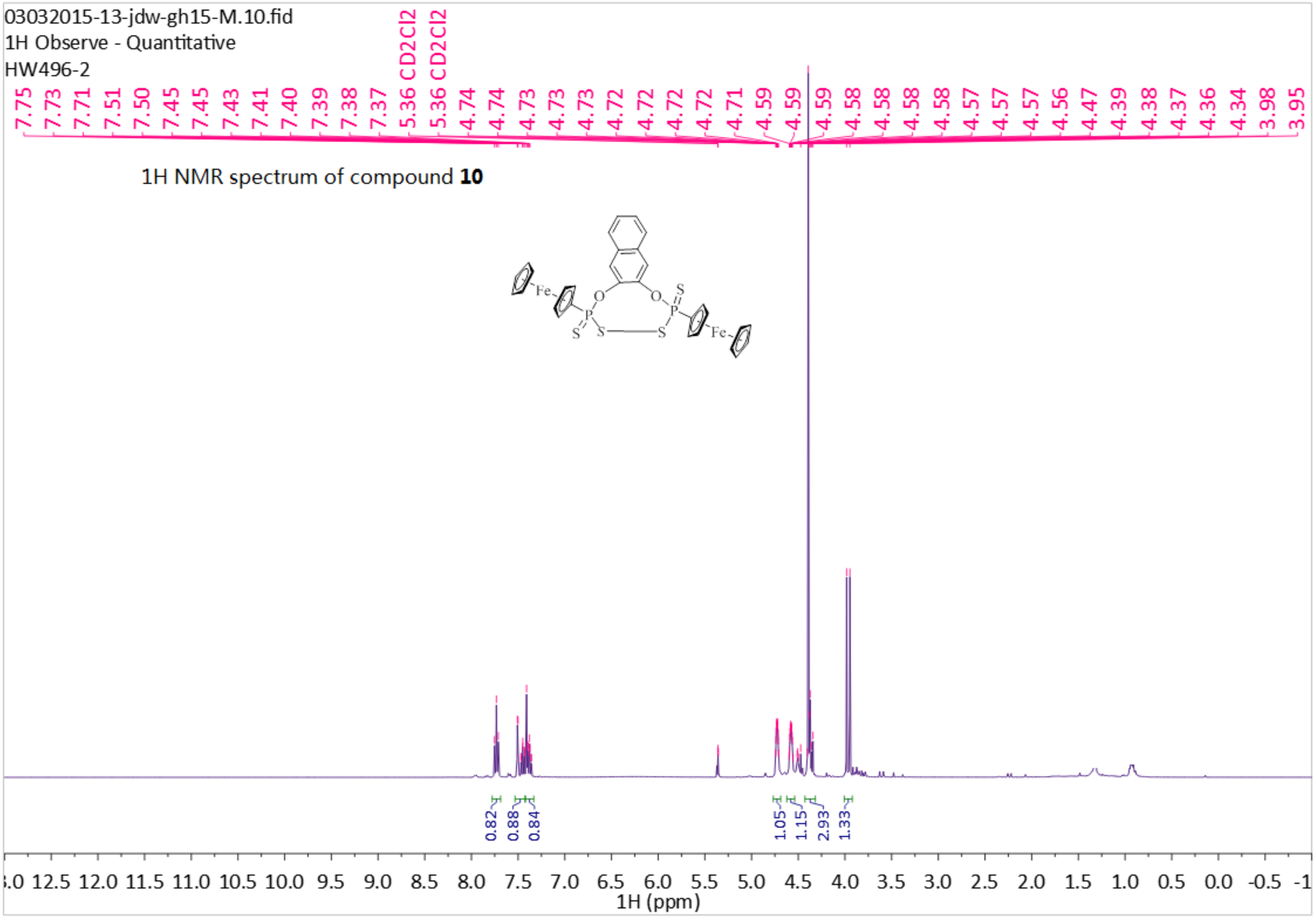
03032015-13-jdw-gh15-M.10.fid
1H Observe - Quantitative
HW496-2
7.75 7.73 7.71 7.51 7.50 7.45 7.45 7.43 7.41 7.40 7.39 7.38 7.37 5.36 CD2Cl2 5.36 CD2Cl2 4.74 4.74 4.73 4.73 4.73 4.72 4.72 4.72 4.72 4.71 4.59 4.59 4.59 4.58 4.58 4.58 4.58 4.57 4.57 4.57 4.56 4.47 4.39 4.38 4.37 4.36 4.34 3.98 3.95
1H NMR spectrum of compound **10**
Fe
O
O
S
P
P
S
S
S
Fe
0.82 0.88 0.84 1.05 1.15 2.93 1.33
1H (ppm)

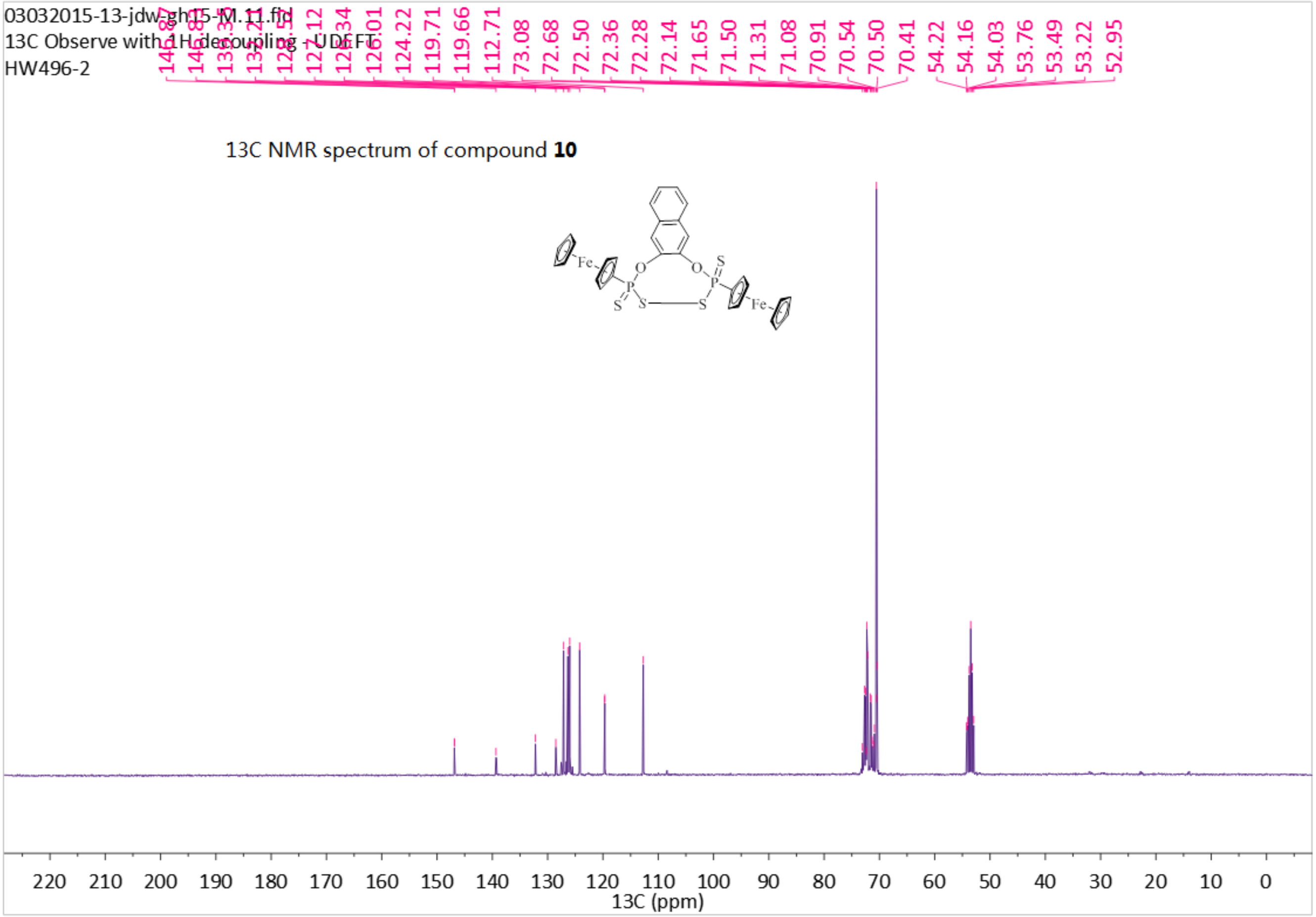
03032015-13-jdw-gh15-M.11.fid
13C Observe with 1H decoupling - UDEFT
HW496-2
13C NMR spectrum of compound **10**
13C (ppm)

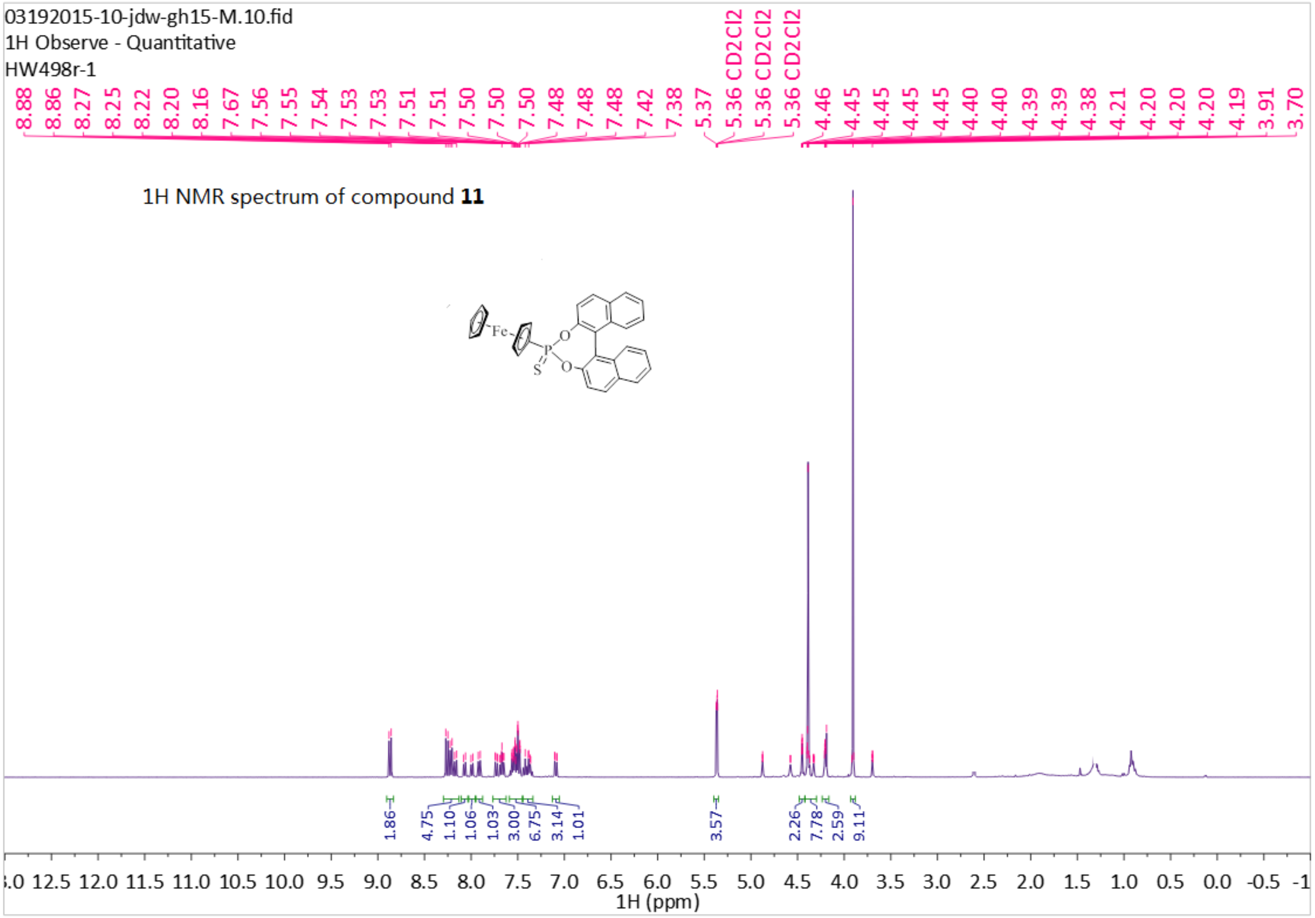
03192015-10-jdw-gh15-M.10.fid
1H Observe - Quantitative
HW498r-1
8.88 8.86 8.27 8.25 8.22 8.20 8.16 7.67 7.56 7.55 7.54 7.53 7.53 7.51 7.51 7.50 7.50 7.50 7.48 7.48 7.48 7.42 7.38 5.37 5.36 CD2Cl2 5.36 CD2Cl2 5.36 CD2Cl2 4.46 4.45 4.45 4.45 4.45 4.40 4.40 4.39 4.39 4.38 4.21 4.20 4.20 4.20 4.19 3.91 3.70
1H NMR spectrum of compound **11**
Fe
P
O
O
S
1.86 4.75 1.10 1.06 1.03 3.00 6.75 3.14 1.01 3.57 2.26 7.78 2.59 9.11
12.5 12.0 11.5 11.0 10.5 10.0 9.5 9.0 8.5 8.0 7.5 7.0 6.5 6.0 5.5 5.0 4.5 4.0 3.5 3.0 2.5 2.0 1.5 1.0 0.5 0.0 -0.5
1H (ppm)

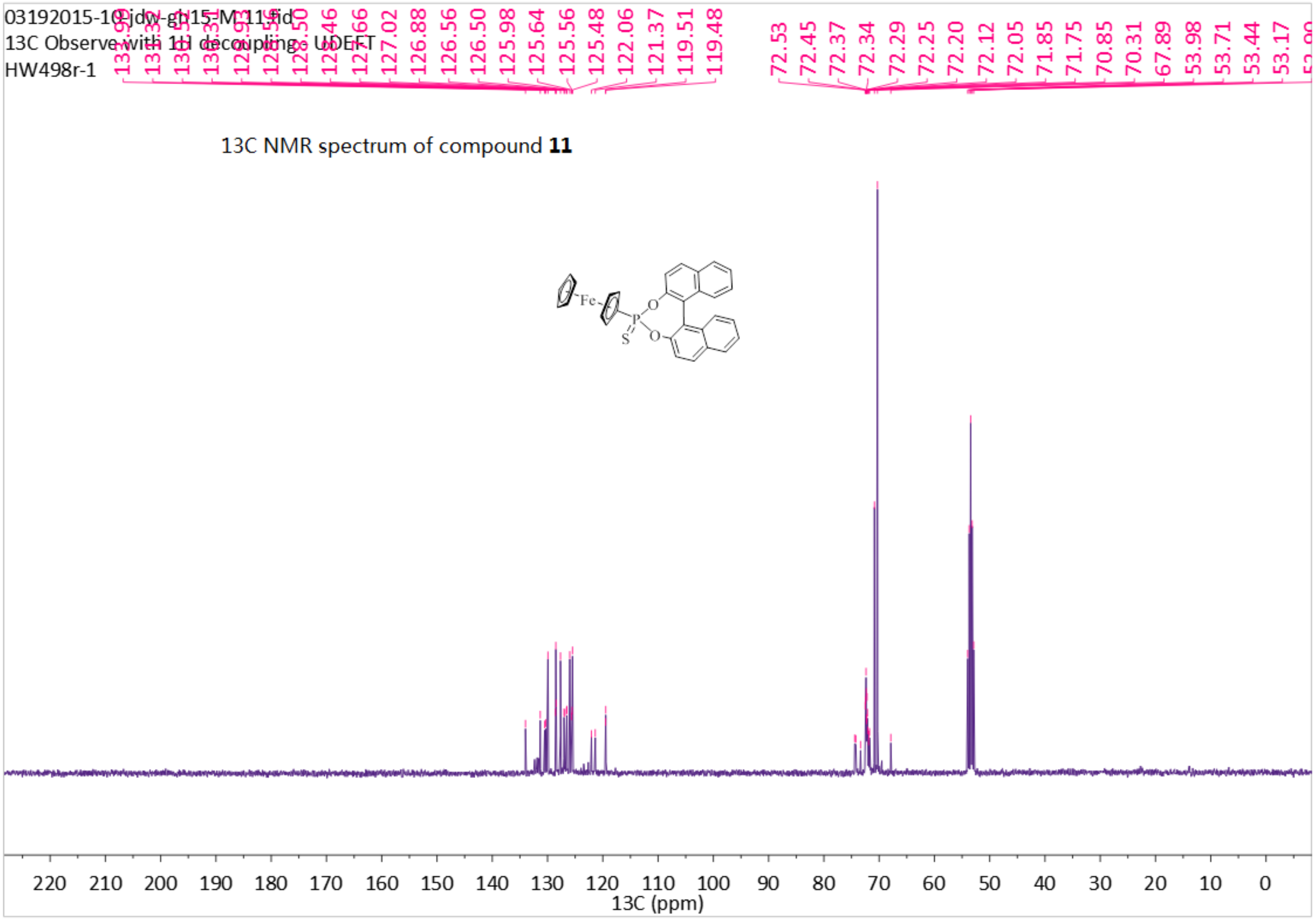
HW498r-1
13C NMR spectrum of compound **11**
127.02
126.88
126.56
126.50
125.98
125.64
125.56
125.48
122.06
121.37
119.51
119.48
72.53
72.45
72.37
72.34
72.29
72.25
72.20
72.12
72.05
71.85
71.75
70.85
70.31
67.89
53.98
53.71
53.44
53.17
220 210 200 190 180 170 160 150 140 130 120 110 100 90 80 70 60 50 40 30 20 10 0
13C (ppm)

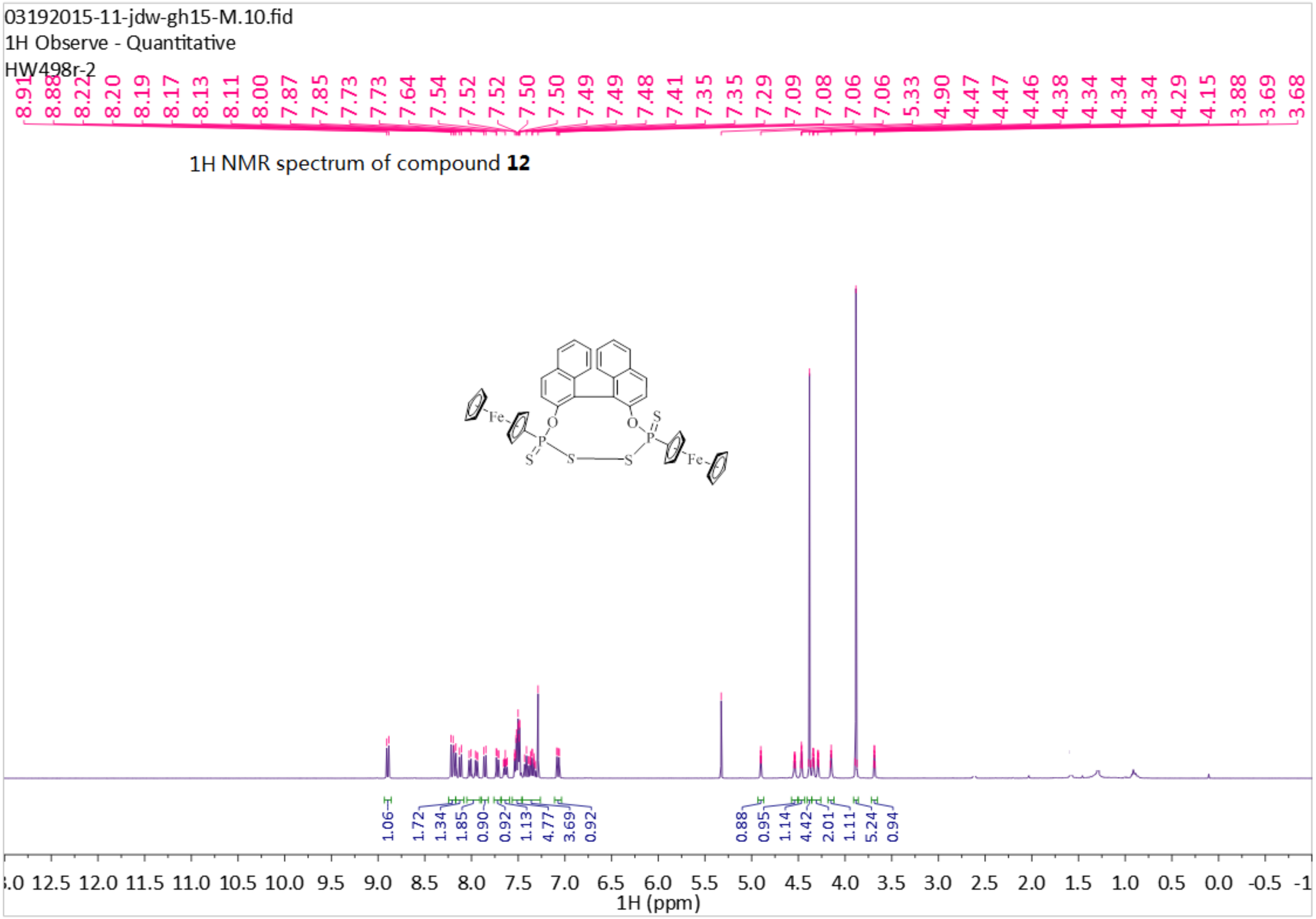
03192015-11-jdw-gh15-M.10.fid
1H Observe - Quantitative
HW498r-2
1H NMR spectrum of compound **12**
1H (ppm)

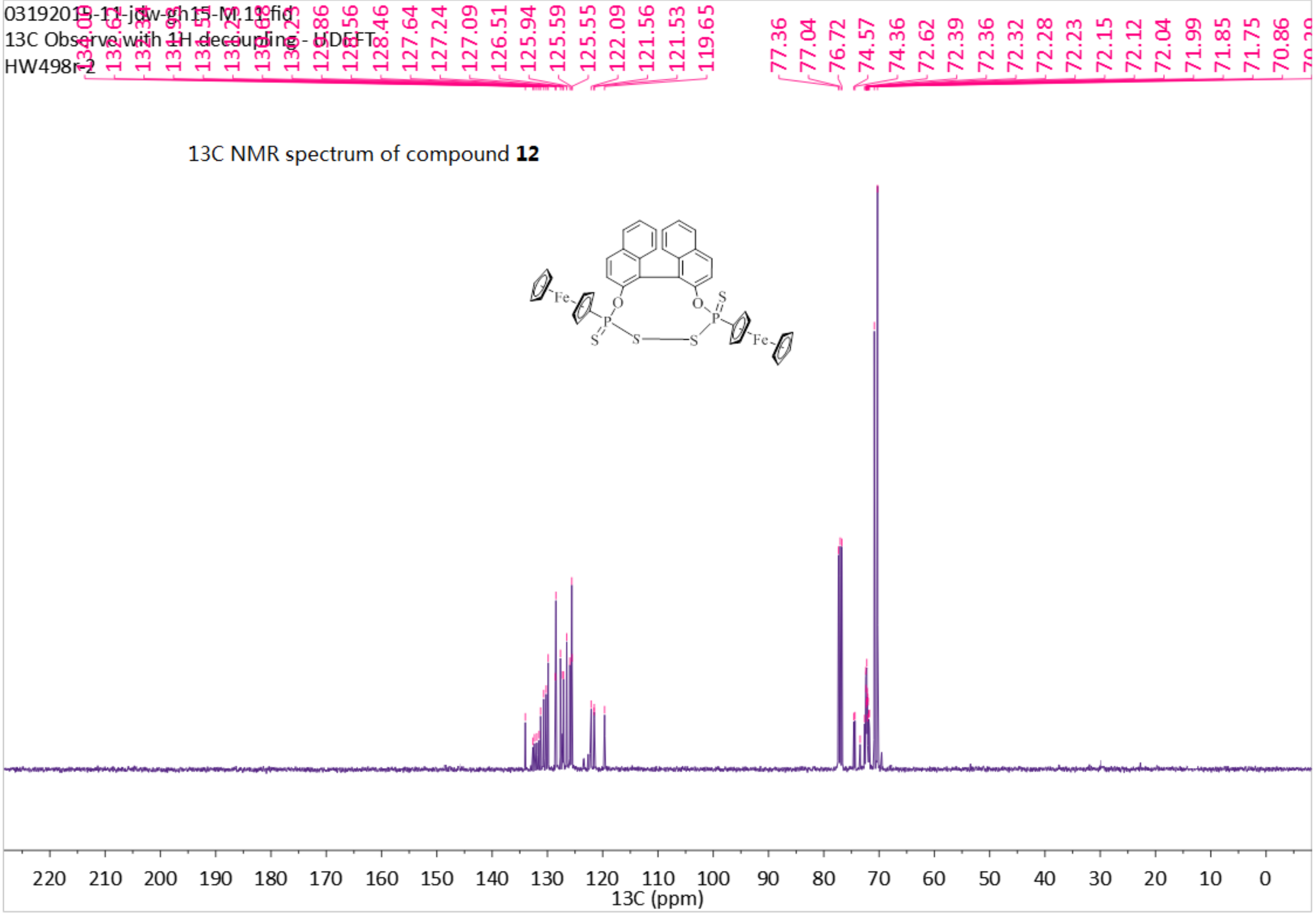

13C NMR spectrum of compound **12**
